# An integrative re-evaluation of *Typhlatya* shrimp within the karst aquifer of the Yucatán Peninsula, Mexico

**DOI:** 10.1038/s41598-022-08779-9

**Published:** 2022-03-29

**Authors:** Lauren Ballou, David Brankovits, Efraín M. Chávez-Solís, José M. Chávez Díaz, Brett C. Gonzalez, Shari Rohret, Alexa Salinas, Arielle Liu, Nuno Simões, Fernando Álvarez, Maria Pia Miglietta, Thomas M. Iliffe, Elizabeth Borda

**Affiliations:** 1 Department of Marine Biology, Texas A&M University at Galveston, 200 Seawolf Pkwy, Galveston, TX USA; 2 Molecular Ecology Group, Water Research Institute, National Research Council of Italy (IRSA CNR), 28922 Pallanza, Italy; 3 Posgrado en Ciencias Biológicas, Unidad de Posgrado, Edificio A, 1er piso, Circuito de Posgrados, Ciudad Universitaria, Coyoacán, Ciudad de México, Mexico; 4 Instituto de Investigaciones Oceanológicas, Universidad Autónoma de Baja California, Ensenada, Baja California Mexico; 5 Department of Invertebrate Zoology, Smithsonian Institution, National Museum of Natural History, P.O. Box 37012, Washington D.C., USA; 6 Department of Earth, Atmospheric and Planetary Sciences, Massachusetts Institute of Technology, Green Bldg., 77 Massachusetts Ave, Cambridge, MA USA; 7 Geology & Geophysics Department, Woods Hole Oceanographic Institution, 266 Woods Hole Road, MS #52, Woods Hole, MA USA; 8 Department of Biological Sciences, University of Notre Dame, 100 Galvin Life Science Center, Notre Dame, IN USA; 9 School of Anthropology, University of Arizona, Emil W. Haury Anthropology Bldg., 1009 E South Campus Dr., Tucson, AZ USA; 10 Unidad Multidisciplinaria de Docencia e Investigación, Facultad de Ciencias, Universidad Nacional Autónoma de México, Puerto de Abrigo S/N, Sisal, Yucatán, Mexico; 11 National Coastal Resilience Laboratory (LANRESC), Puerto de Abrigo S/N, Sisal, Yucatán, Mexico; 12 International Chair for Ocean and Coastal Studies in Mexico, Harte Research Institute, Texas A&M at Corpus Christi, 6300 Ocean Drive, Corpus Christi, TX USA; 13 Colección Nacional de Crustáceos, Instituto de Biología, Universidad Nacional Autónoma de México, A.P. 70-153, 04510 Coyoacán, México D.F. Mexico; 14 Department of Life Sciences, Texas A&M University San Antonio, One University Way, San Antonio, TX USA

**Keywords:** Evolutionary genetics, Phylogenetics, Taxonomy, Evolutionary biology, Biodiversity, Conservation biology

## Abstract

The Yucatán Peninsula, Mexico is a carbonate platform well-known for extensive karst networks of densely stratified aquifer ecosystems. This aquifer supports diverse anchialine fauna, including species of the globally distributed anchialine shrimp genus *Typhlatya* (Atyidae). Four species (*T. campecheae*, *T. pearsei*, *T. dzilamensis* and *T. mitchelli)* are endemic to the Peninsula, of which three are federally listed in Mexico. This first integrative evaluation (i.e., molecular, morphological, broad geographic and type locality sampling, and environmental data) of Yucatán *Typhlatya* reveals considerable species identity conflict in prior phylogenetic assessments, broad species ranges, syntopy within cave systems and five genetic lineages (of which two are new to science). Despite sampling from the type locality of *endangered T. campecheae*, specimens (and molecular data) were indistinguishable from *vulnerable T. pearsei*. Ancestral/divergence reconstructions support convergent evolution of a low-salinity ancestor for a post-Paleogene arc Yucatán + Cuba *Typhlatya* clade within the anchialine Atyidae clade. A secondary adaptation for the coastal-restricted euryhaline (2–37 psu), *Typhlatya dzilamensis* (unknown conservation status) was identified, while remaining species lineages were low-salinity (< 5 psu) adapted and found within the meteoric lens of inland and coastal caves. This study demonstrates the need for integrative/interdisciplinary approaches when conducting biodiversity assessments in complex and poorly studied aquifers.

## Introduction

**Figure 1:**
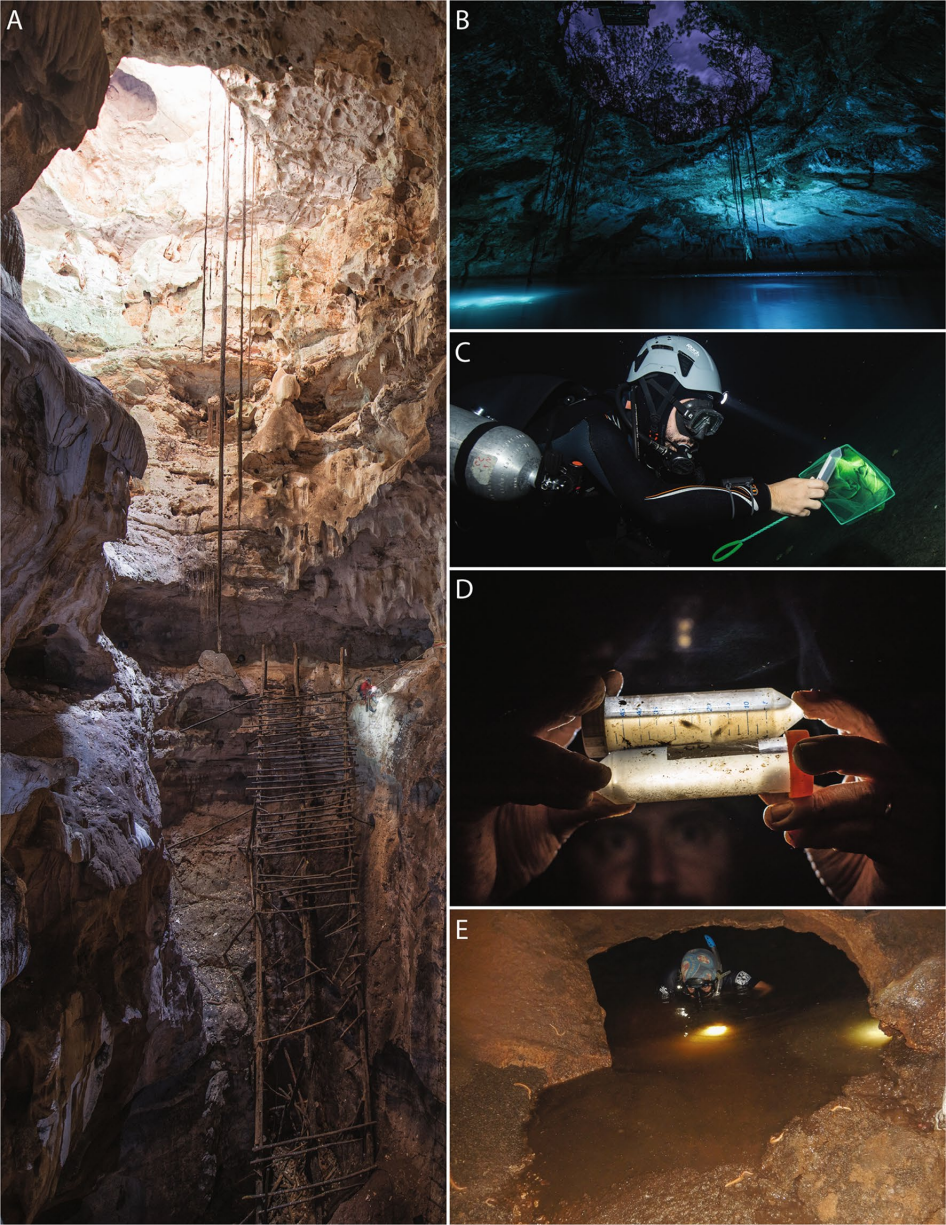
Select field sites and sampling of *Typhlatya* specimens within the Yucatán Peninsula. (**A**) Entrance to Grutas de Xtacumbilxunam (Campeche State), showing pit-style inland *cenote*; depth ~ 60 m; note replica of wooden ladder used by Mayans to access aquifer; (**B**) Cenote Noh-Mozon (Yucatán State), inland cave, and collection site of *Typhlatya pearsei*; (**C**) Cave diver (ECS) collection of *Typhlatya mitchelli* within Cenote Tza Itza (inland, Yucatán State) using catch net and falcon tube; (**D**) post collection (by ECS) of *Typhlatya campecheae* within Grutas de Xtacumbilxunam, type locality; (**E**) Cave diver (ECS) emerging from freshwater entrance pool within Grutas de Xtacumbilxunam; collection site of *Typhlatya campecheae*. Images: (**A–D**) Kay Vilchis; (**E**) Erick Sosa.

**Figure 2:**
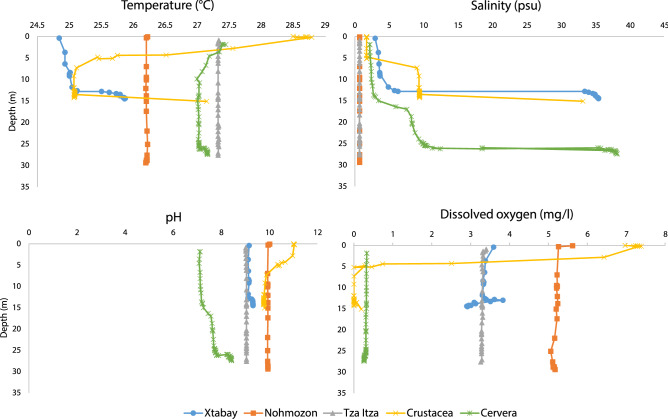
Vertical profile of physical and chemical hydrological parameters (Temperature, Salinity, pH and Dissolved Oxygen) of Cave Systems, Xtabay (blue), Nohmozon (orange), Tza Itza (grey), Crustacea (yellow) and Cervera (green). Data obtained using a Hydrolab DataSonde5.

The Yucatán Peninsula, Mexico consists of an emerged carbonate platform spanning 165,000 km^2^ that contains extensive karst aquifer ecosystems^1^ with networks of inland and coastal caves accessed via *cenotes* (local term for sinkholes)^2–4^. The Peninsula’s unconfined aquifer has density stratification where marine origin saline groundwater mixes with the meteoric lens^5–8^. Consequently, the flooded caves are characterized by meromictic conditions with well-defined haloclines that occur progressively deeper from coastal to inland regions^4,7,9,10^ Cave entrances are generally pit-style formations (e.g., Fig. 1A, B) formed via terrestrial collapse, leading to subterranean passages that branch into extensive anastomotic networks^3,10^. Biogeochemical cycling associated with the haloclines have a vital role in sustaining the subterranean food web^11^. The spatial variability in salinity and hydrogeology (Fig. 2) creates a collection of coastal subterranean estuaries^5,12^ that host a diverse array of characteristic stygobitic (aquatic cave-adapted) fauna^13–15^. These environments and associated fauna are also widely referred to as anchialine^16–18^. How these spatial variables shape the biodiversity and evolution of anchialine fauna remains poorly understood.

More than 170 anchialine species have been recorded in the Yucatán Peninsula, the majority of which are crustaceans^13–15,19^. Species of the atyid shrimp genus *Typhlatya* Creaser, 1936 (Atyidae De Haan, 1849 (in De Haan, 1833–1850)) were among the first cave fauna described from the Yucatán Peninsula^20^. The genus consists of 17 species and exhibits a globally disjunct distribution within anchialine habitats: Yucatán Peninsula, Caribbean (Puerto Rico, Dominican Republic, Lesser Antilles, Netherlands Antilles, Columbia, Honduras), West Indies (Bahamas, Turks and Caicos), Bermuda, Ascension Island, Mediterranean (France, Spain), and the Galapagos Islands; with one undescribed species from Zanzibar^21,22^. The placement of *Typhlatya arfeae* Jaume & Bréhier, 2005, *Typhlatya galapagensis* Monod & Cals, 1970, *Typhlatya miravetensis* Sanz & Platvoet, 1995, and *Typhlatya monae* Chace, 1954 within *Typhlatya* is debated, as recent phylogenetic work suggests these species are more closely related to *Antecaridina* Edmondson, 1954, *Halocaridina* Holthuis, 1963, *Stygiocaris* Holthuis, 1960, and/or *Typhlopatsa* Holthuis, 1956; resulting in the paraphyly of *Typhlatya*^21,22^.

**Figure 3:**
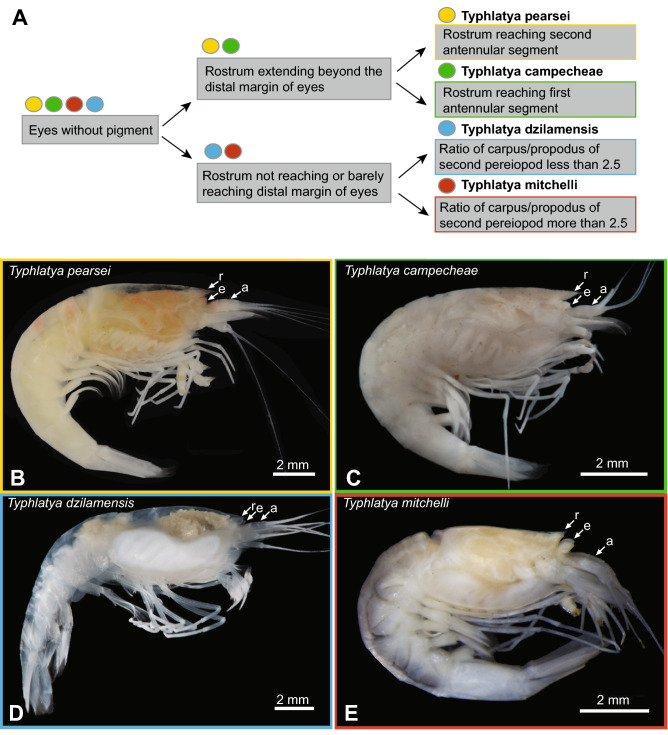
(**A**) Graphical morphological key of *Typhlatya* spp. of the Yucatán Peninsula adapted from Alvarez et al.^25^; (**B–E**) Species of the Yucatán Peninsula. (**B**) *Typhlatya pearsei *(‘Ultimo Suspiro’, near Dzilam de Bravo); (**C**) *Typhlatya campecheae* (Grutas de Xtacumbilxunam; ID = C2*); (**D**) *Typhlatya dzilamensis* (Cenote Crustacea); (**E**) *Typhlatya mitchelli* (Sistema Paamul; ID = TI008D*). *a*, Antennular peduncle; *e* eyes, *r* rostrum. Images taken by ECS, AL, SR, and AS. *Genetically identified in this study, Fig. 4.

To date, four species of *Typhlatya* have been described from the Yucatán Peninsula: *Typhlatya pearsei* Creaser, 1936, *Typhlatya campecheae* H.H. Hobbs III & H.H. Hobbs Jr., 1976, *Typhlatya mitchelli* H.H. Hobbs III & H.H. Hobbs Jr., 1976, and *Typhlatya dzilamensis* Álvarez, Iliffe & Villalobos, 2005. Three of the four species are federally listed as *vulnerable* (*T. pearsei*, *T. mitchelli*) or *endangered* (*T. campecheae*)^23^. Traditional species descriptions primarily utilized morphological differentiation, illustrations, and locality data as diagnostic^20,24,25^. While each species is currently accepted as morphologically distinguishable (Fig. 3), character variability has been observed in *T. pearsei*^26^. Because these records came from a time before molecular techniques were easily accessible and widely used as standard practice, no molecular data is available for type species. The lack of molecular data from type material, the potential for cryptic speciation, and variable morphology poses challenges in consistent identifications of subterranean crustacean species^27–29^.

**Figure 4:**
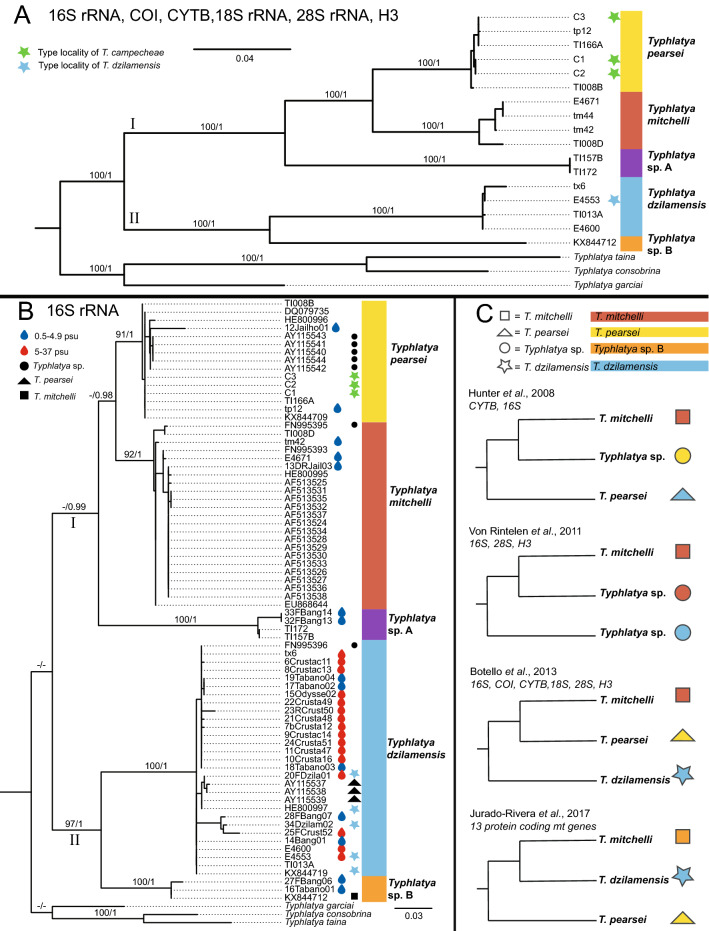
Phylogenetic hypotheses of *Typhlatya* spp. from the Yucatán Peninsula. (**A**) Multi-gene (16S rRNA, COI, CYTB, 18S rRNA, 28S rRNA, H3) phylogeny of select *Typhlatya* spp. sequences, Bayesian phenogram shown; (**B**) 16S rRNA phylogeny of GenBank and sampling new to this study, Bayesian phenogram shown; (**C**) Graphical summary of phylogenetic relationships from previous studies^21,22,30,31^. Support values indicated at nodes above branches: Bootstrap/Bayesian Posterior Probability. Supports values < 90/0.90, not shown (see text) and indicated by ‘–’. Colors represent species identity resulting from the molecular evaluations of this study (**A–C**): *Typhlatya pearsei* = yellow, *Typhlatya mitchelli* = red, *Typhlatya dzilamensis* = blue, *Typhlatya* sp. A purple, *Typhlatya* sp. B orange. Filled black shapes beside species’ identifications (**B**) indicate previous identifications of GenBank material in conflict with the present study: circle = *Typhlatya sp.,* triangle = *T. pearsei*, square = *T. mitchelli.* Shapes beside species’ identifications (**C**) indicate species identifications from previous work: circle = *Typhlatya* sp., triangle = *T. pearsei*, square = *T. mitchelli*, star = *T. dzilamensis*. Sequences from specimens collected from type localities indicated by colored stars (**A,B**): green star = *T. campecheae*, Grutas de Xtacumbilxunam; blue star = *T. dzilamensis,* Dzilam de Bravo*.* Sequences from samples with known salinity (in psu) are indicated by colored water droplets (**B**), blue = low salinity, 0.5–4.99, red = high salinity, 5–37.

In recent years, many studies have sought to understand the evolutionary history and relationships within *Typhlatya* and its closest relatives^21,22,29–34^. These investigations have produced useful genetic data (mitochondrial and nuclear genes) for Yucatán Peninsula *Typhlatya* species and made available via GenBank^35^. However, it is not uncommon to find that publicly available sequences have erroneous identifications leading to taxonomic and systematic confusion^36–40^. What prompted the current study was an evaluation of data from previous work, which revealed conflicting species identifications and incongruent phylogenetic hypothesis^21,22,29–34^. Therefore, the objective of this study was to conduct the first integrative biodiversity assessment within the poorly studied karst aquifer of the Yucatán Peninsula through the evaluation of molecular (published and newly sequenced) data, morphological data, sampling from broad geographic and key type localities, and environmental/salinity data, in order to clarify species identities and infer the evolutionary history and distribution of Yucatán *Typhlatya* clade members.

## Results

The phylogenetic relationships of *Typhlatya* specimens were inferred from 16S rRNA (16S; 74 sequences, 515 bp), Cytochrome c oxidase subunit I (COI; 54 sequences, 1233 bp), Cytochrome b (CYTB; 49 sequences, 588 bp), 18S rRNA (18S; 15 sequences, 1779 bp), 28S rRNA (28S; 21 sequences, 1090 bp) and Histone 3 (H3, 21 sequences, 327 bp). Maximum Likelihood (ML) and Bayesian Inference (BI) reconstructions based on a concatenated multi-gene dataset (16S, COI, CYTB, 18S, 28S and H3; Total: 5514 bp) and 17 Yucatán *Typhlatya* individuals, supported two main clades (I and II) and five subclades (Fig. 4A), each represented by *T. pearsei*, *T. mitchelli*, *T. dzilamensis*, and two unknown lineages, the latter hereafter, referred to as *Typhlatya* sp. A and *Typhlatya* sp. B; see details below.

Species delimitation analyses provide support for five lineages within the Yucatán Peninsula (STable 1). In all single-gene and concatenated GMYC analyses, a minimum of five phylogenetic entities were recovered with significant support (p < 0.05) with confidence interval ranging between 3 and 16. A minimum of four to six entities were recovered within bPTP analyses and five to nine within PTP analyses. Both *Typhlatya* sp. A and *Typhlatya* sp. B were recovered as their own entities throughout each GMYC analysis, providing support for two new independent lineages within the Yucatán Peninsula. PTP recovered, in both the COI and concatenated datasets, 2–3 independent lineages within the *Typhlatya* sp. A and *Typhlatya* sp. B complexes. Additionally, all individuals sampled from the type locality of *T. campecheae* were recovered within the *T. pearsei* lineage in each analysis. Inter-clade genetic uncorrected pairwise (p)-distances of 16S and COI ranged 5.40–18.1% and 10.0–19.5%, respectively. Intra-clade uncorrected p-distances was highest in COI (3.5%) and 16S (1.2%) for *Typhlatya* sp. A and less than 2.0% for remaining clades in both genes. See STable 2 for summary of intra- and inter-clade uncorrected p-distances.

### Phylogeny of the Yucatán clade

ML and BI analyses of six concatenated genes (6 Partitions: (1)COI + CYTB, 1st Codon/(2)COI + CYTB, 2nd Codon/(3)COI + CYTB, 3rd Codon/(4)16S + H3, 3rd Codon/(5) 18S + H3 1st Codon/(6) 28S + H3, 2nd Codon) recovered two main clades within *Typhlatya*, each with fully supported subclades (ML Bootstrap, BS 100; Bayesian Posterior Probability, BPP 1), and non-conflicting topologies between analyses: Clade I, *Typhlatya* sp. A + (*T. mitchelli* + *T. pearsei*) and Clade II, *T. dzilamensis* + *Typhlatya* sp. B, respectively (Fig. 4A). Individuals (C1–3; n = 3) sampled from the type locality of *T. campecheae* (Grutas de Xtacumbilxunam, Campeche State (CS)) were recovered within the *T. pearsei* clade. *Typhlatya* sp. A was represented by individuals sampled from Cenotes Nah-Yah (Yucatán State (YS); n = 1) and Kankirixche (YS; n = 1), and recovered as sister to *T. pearsei* + *T. mitchelli*. *Typhlatya* sp. B sampled from Cenote Hoctún (YS; n = 1) was recovered as sister to *T. dzilamensis*. The *T. dzilamensis* clade was comprised of an individual (E4553) from the type locality (Dzilam de Bravo, YS) of this species, as well as individuals (n = 3) from Cenotes Actun Ha (Quintana Roo State, QRS), Sabtun 1 (YS), and X’tabay (YS). Remaining sampling localities are listed in STable 3.

ML and BI analyses of 16S (Fig. 4B) recovered congruent topologies to the multi-gene hypothesis (Fig. 4A), except for the outgroup taxon *T. garciai*, which was recovered as sister to Clade II (*T. dzilamensis* + *Typhlatya* sp. B) in the BI analysis, however, with low support (BPP 0.66). Like the multi-gene phylogeny, two main clades were recovered: Clade I (BS 89, BPP 0.99), *Typhlatya* sp. A + (*T. mitchelli* + *T. pearsei;* BS 72, BPP 0.98), and Clade II (BS 97, BPP 1), *T. dzilamensis* + *Typhlatya* sp. B.

Single-gene analyses of 28S, COI, and H3 recovered congruent topologies with the multi-gene hypothesis, although *Typhlatya* sp. B was absent from 28S and H3 analyses (SFigs. 1–3). The analysis of COI included unpublished sequence data analyzed previously in an undergraduate thesis^41^, which were congruent with the multi-gene and 16S topologies. COI data from the type locality of *T. campecheae* was not attained for this study (to be revisited elsewhere; Vaughn et al., in prep), however a representative from Cenote Cantemo (Cantemo, Campeche; ~ 100 km south of Grutas de Xtacumbilxunam) indicated some divergence (~ 5% uncorrected pairwise distance) from remaining *T. pearsei* representatives but was well nested within the *T. pearsei* clade. Within CYTB analyses, one Genbank sequence (HE800951) was excluded due to limited overlap with other sequences in the alignment. Analyses of 18S yielded a slightly incongruent topology, with *Typhlatya* sp. A recovered as sister to remaining Yucatán *Typhlatya* species (BS 77, BPP 0.98), while data for *Typhlatya* sp. B was absent, and thus not represented in the topology (SFigs. 1–3).

### Genetic (mis)identities of Typhlatya

The phylogenetic hypotheses (Fig. 4A,B, SFigs. 1–3), including published and newly sequenced data, were incongruent with previous hypotheses and presented conflicts in species identification, except for Botello et al*.*^21^ (in part); see Fig. 4C and STable 4. GenBank sequences identified as *T. mitchelli* (n = 30) were overall consistent, except for one representative (ZMB DNA-600^22^), which was recovered as *Typhlatya* sp. B. GenBank sequences attributed to *T. pearsei* (n = 7) were consistent in identity, except for four representatives (27-1^31,34^, SM6^31,34^, AA3^31,34^ and Zaksek et al.^29^), which instead were nested within the *T. dzilamensis* clade. GenBank sequences identified as *Typhlatya* sp. (n = 7) were recovered (and revised) as followed: *T. mitchelli* clade*,* ZMB DNA-603^31^; *T. pearsei* clade, SAY 2-6^30,34^; and *T. dzilamensis* clade, ZMB DNA-604^31^. Lastly, individuals sampled from the type locality of *T. campecheae* (Grutas de Xtacumbilxunam; n = 3) and non-type locality Cenote Cantemo (Campeche; n = 1) were recovered within the *T. pearsei* clade, while individuals sampled from the type locality (previously^21,22^ and in this study) of *T. dzilamensis* (Dzilam de Bravo; n = 6) were recovered within the *T. dzilamensis* clade.

In total, five misidentifications were uncovered from GenBank data (summarized in Fig. 4C, STable 4): (i) *Typhlatya* sp. (sensu Hunter et al.^30^—CYTB, 16S ), here recovered as *T. pearsei*; (ii) *T. pearsei* (sensu Hunter et al.^30^—CYTB, COI, 16S; Zakšek et al.^29^—28S), here recovered as *T. dzilamensis*; (iii) *Typhlatya* sp*.* (sensu von Rintelen et al.^31^—16S, H3, 28S), here recovered as *T. mitchelli*; (iv) *Typhlatya* sp*.* (sensu von Rintelen et al.^31^—16S, H3, 28S), here recovered as *T. dzilamensis*; and (v) *T. mitchelli* (sensu Jurado-Rivera et al*.*^22^—CYTB, COI, 16S), here recovered as *Typhlatya* sp. B*.* Each clade showed conflicting identities, but the highest number was found for *T. dzilamensis* and *T. pearsei*.

### Species distributions

**Figure 5:**
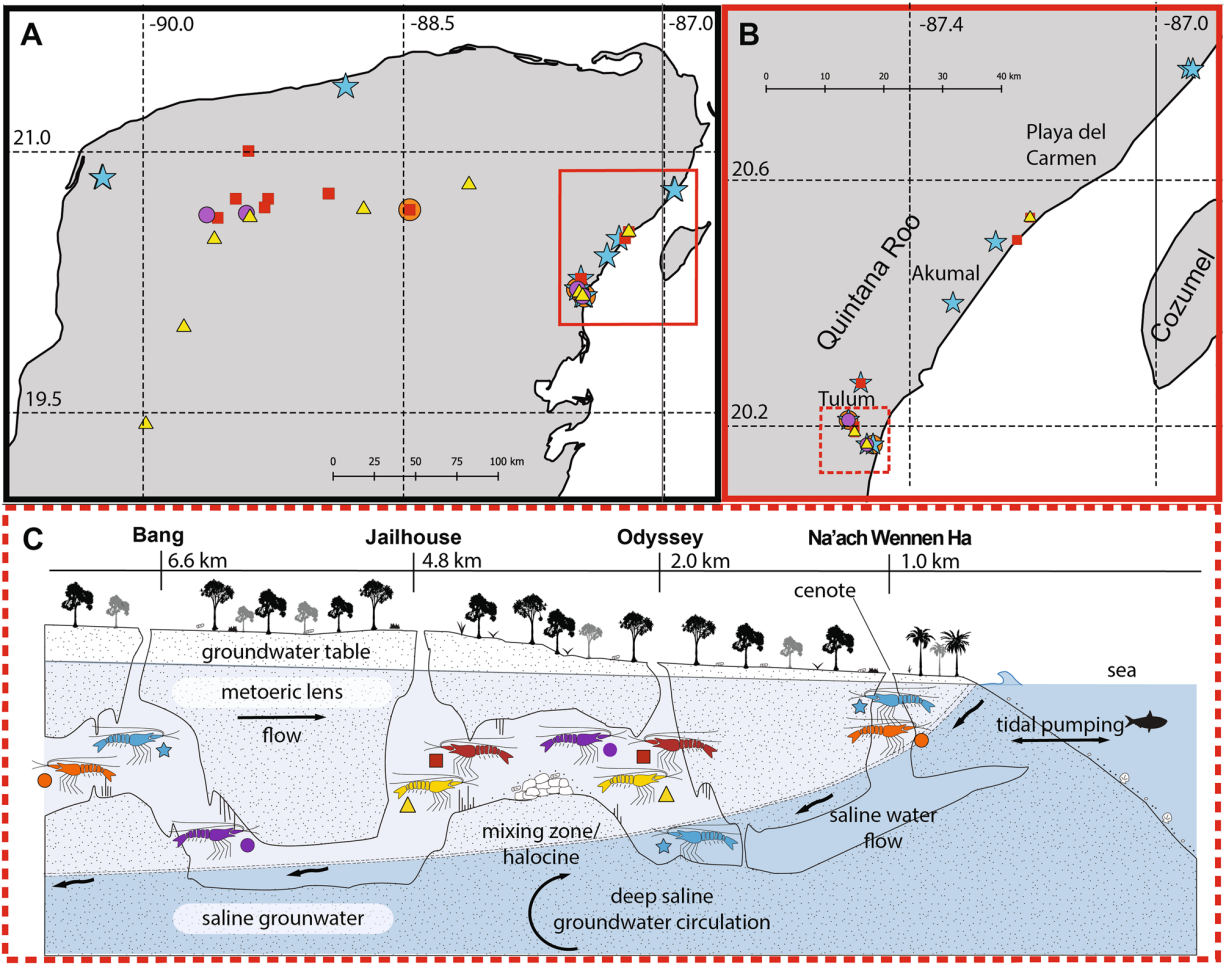
Geographic distribution of *Typhlatya* spp. included in this study. (**A**) Sampling distribution of *Typhlatya* spp. (Yucatán Peninsula); (**B**) sampling distribution of *Typhlatya* spp. (Quintana Roo State, QRS); (**C**) hypothetical cave profile of the Ox Bel Ha system (QRS) and schematic sampling distribution of the five species identified in this study, collected by salinity layer. Shrimp marked with an asterisk indicate unknown salinity values. Colored symbols indicate species identification: *Typhlatya pearsei* = yellow, triangle; *Typhlatya mitchelli* = red, square; *Typhlatya dzilamensis* = blue, star; *Typhlatya* sp. A = purple, circle; *Typhlatya* sp. B = orange, circle. Cave profile (**C**) adapted from Brankovits et al.^11^ and modified by LB and EB. Maps were made in QGIS using metadata from Natural Earth.

Evaluation of the genetic identities of *Typhlatya* specimens using 16S, COI and multi-gene phylogenetic approaches supported broad ranges for each of the five identified species lineages among caves. A total of 109 *Typhlatya* individuals were observed within 31 inland and coastal caves (Fig. 5) from variable salinities (0.5–37 practical salinity unit, psu; STable 3), here also including reassigned GenBank sequence data (Fig. 4, STable 4). *Typhlatya mitchelli* (n = 36; *cenotes* = 13) was observed inland (YS), as well as in coastal caves (QRS). This species was sampled from the meteoric lens (‘low salinity’ waters; < 4.99 psu), above the halocline, within Cenotes Odyssey (OBH, QRS), Jailhouse (2.9 psu; OBH, QRS) and Tza Itza (0.63 psu; YS). *Typhlatya pearsei* (n = 19; *cenotes* = 9) was found inland (YS, CS) and in coastal (QRS) caves in the meteoric lens, above the halocline, within Cenotes Jailhouse (3.5 psu; OBH, QRS), Noh-Mozon (0.7 psu; YS), Grutas de Xtacumbilxunam (CS) and Cantemo (CS).

*Typhlatya dzilamensis* (n = 41; *cenotes* = 11) was observed in the north/north-western coastal YS and QRS caves, spanning ~500 km. This species was recorded from Cenotes Sabtun 1 (YS) and Santa Maria (YS), with the greatest distance ~ 17–18 km inland from the coastline. *Typhlatya dzilamensis* was the only species sampled from both above and below the halocline in salinities ranging from oligohaline to saline. Within the OBH system (QRS), *T. dzilamensis* was observed from multiple salinities, above (oligohaline) and/or below (saline) the halocline(s), while in Cenote Crustacea (QRS; Fig 2), within mesohaline and saline waters. *Typhlatya* sp. A (n = 7; *cenotes* = 4) was observed within inland (Kankirixche and Nah-Yah, YS) and coastal (Odyssey and Bang, 2.5 psu; OBH, QRS) caves, and sampled above the halocline in oligohaline waters. *Typhlatya* sp. B (n = 6; *cenotes* = 4) was also found within inland (Hoctun, YS) and coastal (Na’ach Wennen Ha and Bang, OBH, QRS) systems, sampled above the halocline in oligohaline waters (Na’ach Wennen Ha and Bang), with one specimen reported from below the halocline (Na’ach Wennen Ha)^41^.

Representatives of all five lineages were collected from the meteoric lens within the OBH cave system (Fig. 5). Specifically, species were collected syntopically from Cenotes Bang (*Typhlatya* sp. A, *Typhlatya* sp. B, *T. dzilamensis*; ~ 2 psu), Jailhouse (*T. mitchelli*, *T. pearsei*; ~ 3 psu), (3.5 psu; OBH, QRS), Odyssey (*T. mitchelli*, *T. pearsei, Typhlatya* sp. A and *T. dzilamensis*); and Na’ach Wennen Ha (*Typhlatya* sp. B, *T. dzilamensis*; ~ 4 psu), respectively. *Typhlatya* species also occurred syntopically within Cenotes Actun Ha (*T. dzilamensis, T. mitchelli*; Tulum, QRS), Hoctún (*Typhlatya* sp. B, *T. mitchelli*; Hoctún, YS), and Sistema Paamul (*T. mitchelli*, *T. pearsei*; Paamul, QRS), respectively.

#### Morphology

*Typhlatya* species were evaluated based on the key developed by Álvarez et al.^25^ (Fig. 3, SFig. 4). *Typhlatya pearsei* and *T. campecheae* were traditionally distinguished by rostrum length relative to antennular segments^25^, with the latter known only from the type locality (i.e., Grutas de Xtacumbilxunam). Newly collected individuals of *T. pearsei* and those collected from caves in Campeche State (i.e., the type locality of *T. campechea*e and Cenote Cantemo) exhibited variable rostrum length, but consistent with the rostrum extending past the eyestalk and towards the end of the first or midway to the second antennular segment (SFig. 4A,B). The rostrum was found to be shorter than the eyestalk in *T. mitchelli*, whereas the rostrum of *T. dzilamensis* extended to and just beyond the eyestalk^25^. The rostrum curved upward in *T. mitchelli*, but not in *T. dzilamensis*, consistent with species comparisons by Álvarez et al.^25^. Like *T. mitchelli*, the rostrum of *Typhlatya* sp. A did not extend beyond the eyes; however, was distinct in that it appeared less robust and flatter than the former, and projecting downwards towards the antennae^41^. *Typhlatya* sp. B was not available for direct morphological evaluation, however, photo-documentation^41^ reported a rostrum length between *T. pearsei* and *T. mitchelli*, extending beyond the eyes but not reaching the second antennular segment. A provisional key based on genetic identification and morphological evaluation stemming from this study, and thus here excludes *T. campecheae* (see details in “Discussion” section) can be found in Supplementary Information (iii. Provisional Key to *Typhlatya* species of the Yucatán Peninsula).

### Divergence dating and ancestral reconstruction

Divergence dating estimates for the Yucatán Clade from the evaluation of the Anchialine Atyidae Clade (Fig. 5) was based on the best fit molecular clock with 5 partitions (excluding the 3rd codon position for COI, CYTB and H3) and 5 independent relaxed log-normal clock models (Yule diversification): (1) COI + CYTB + H3, 1st codon; (2) COI + CYTB, 2nd codon; (3) 16S; (4) 28S; (5) 18S + H3, 2nd codon; see also STable 5–6). The inclusion of a secondary calibration point for the TST complex set to ~ 136 Mya^22^ resulted in older date estimations (STables 5–6; SFig. 5), overall, but well within the Cenozoic for the diversification of the Yucatán clade. Here we focus the discussion of the results as it relates to the Yucatán clade (Table 1, STables 5–6) without this calibration point; see also Discussion.

**Figure 6:**
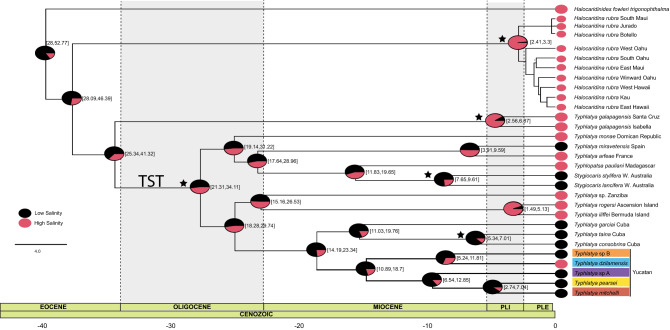
Chronogram showing divergence estimates (in brackets; Mya, 95% high posterior density limits (HPD) as confidence intervals), based on a relaxed, lognormal clock, Yule diversification model, 5 gene partitions (COI + CYB + H3, 1st codon/COI + CYB, 2nd codon/16S/28S/18S + H3, 2nd codon) and 5 independent clock models for the anchialine Atyidae clade. Stars at nodes indicate calibration points used in BEAST2 analyses. Stochastic mapping of salinity trait evolution is visualized with the probability density of each state at internal nodes. Equal rates and unequal estimation (i.e., 0.75 likelihood of a low salinity ancestor) was assumed at the root. Circles at tips denote salinity preferences of lineages, low salinity (black; state 1) and high salinity (red; state 2). Uncertainty of ancestral states indicated at nodes.

**Table 1:** Comparison of mcraAges of Yucatán and Cuba clades estimated in previous work^21,22^ to those of the current study, resulting from BEAST2 analyses.

Clade	Geological		TST + All		Jurado et al. (2017)^22^		Botello et al. (2013)^21^	
	Height		Height		Height		Height	
	Mean	HPD95%	Mean	HPD95%	Mean	HPD95%	Mean	HPD95%
Yucatán *Typhlatya*	14.7	18.69–10.89	39.52	53.27–27.05	56.50	70.07–43.11	14.91	20.22–9.70
*Typhlatya* sp. A + *T. mitchelli* + *T. pearsei*	9.56	12.85–6.544	23.76	34.21–12.44	–	–	–	–
*T. mitchelli* + *T. pearsei*	4.81	7.04–2.74	11.75	18.67–5.67	–	–	4.70	6.90–2.70
*T. dzilamensis* + *Typhlatya* sp. B	8.49	11.81–5.23	22.54	34.11–11.59	33.35	44.89–22.36	–	–
*T. garciai* + (*T. taina* + *T. consobrina*)	15.25	19.76–11.03	39.49	58.12–20.66	–	–	–	–
*T. taina* + *T. consobrina*	6.15	7.01–5.34	6.35	7.24–5.49	43.52	58.66–29.25	5.52	5.99–5.05
Cuba + Yucatán *Typhlatya*	18.57	23.34–14.19	50.9	68.25–34.78	–	–	20.12	26.54–14.24
*T. garciai* + (Cuba + Yucatán *Typhlatya*)	14.7	18.69–10.89	39.52	53.27–27.05	74.01	88.61–60.08	–	–
*T. garciai* + (Yucatán *Typhlatya*)	9.56	12.85–6.544	23.76	68.25–34.21	69.29	82.62–55.40	–	–

Results are based on the relaxed, log-normal clock model with five gene partitions and five independent clocks with four geological events (Geological)^21^ and/or a secondary fossil calibration-based node age for the TST complex (TST+All with Geological) as calibration points^22^.

See also STables 5 and 6 for complete results of all analyses.

The Yucatán clade (14.7 Mya; HPD95% = 18.7–10.9 Mya) was nested within a Late Cenozoic clade (29.7–18.3 Mya), the latter of which comprised of *Typhlatya* species from Zanzibar, Ascension Island, Bermuda, Cuba, and Yucatán Peninsula. Alternatively, *Typhlatya garciai* from the West Indies (Caicos Island) belongs to this clade, but was excluded in the primary analysis due to limited/missing data (SFig. 6). Yucatán Clade I (*T. mitchelli*, *T. pearsei*, *Typhlatya* sp. A) was estimated to have diverged 9.6 Mya (HPD95% = 6.5–12.9 Mya) and Clade II (*T. dzilamensis*, *Typhlatya* sp. B) at 8.5 Mya (HPD95% = 5.2–11.8 Mya). Sister to the Yucatán clade was the Cuban clade comprised of *T. garciai* + (*T. consobrina* + *T. taina*) (15.2 Mya; HPD95% = 19.8–6.3 Mya), with subclade *T. consobrina* + *T. taina* diverging 6.1 Mya (HPD95% = 7.0–5.3 Mya). Details of alternative partition schemes, clock models and comparison to divergence estimates with previous work^22,23^ are summarized in Table 1 and in STables 5–7.

Within stochastic mapping, neither equal nor unequal rates models were found to be more optimal (AIC values 27.8 and 2.8, respectively) and therefore the simplest model (equal) was selected. Within the TST clade, seven of the 17 lineages were found within high salinity waters (> 5 psu; red circles). High salinity lineages were interspersed throughout the TST clade, suggesting that multiple salinity transitions may have occurred, although the majority of deeper ancestral nodes within the clade suggest a low salinity ancestor (<5 psu). *Stygiocaris* spp. and the Yucatán + Cuba clade, in particular, were each recovered with high posterior probability of low salinity ancestors. An independent salinity transition (i.e., *Typhlatya dzilamensis*, low to high salinity/euryhaline) was recovered within the Yucatán + Cuba clade.

## Discussion

### Typhlatya within the Yucatán Peninsula

This study is the first to phylogenetically evaluate both previously published sequences and newly sequenced data across three mitochondrial and three nuclear genes, along with morphological, locality, and salinity data, for *Typhlatya* species sampled across the Yucatán Peninsula. Previous studies collected new molecular data without also evaluating published data in their assessments, resulting in phylogenetic and taxonomic conflict^21,22,31^. A major source of confusion stemmed from the identification of *T. pearsei* and *T. dzilamensis*, given the assumed broad distribution of *T. pearsei* (also incacurately reported from fresh and saline waters^15^) and the unknown salinity preferences of *T. dzilamensis*. Studies that correctly identified *T. dzilamensis* were sampled from the type locality, Dzilam de Bravo (YS)^21,22^. In contrast, *T. dzilamensis* specimens misidentified as *T. pearsei* or *Typhlatya* sp. were sampled from caves beyond the type locality and where salinities were not reported, or not a focus^29–31^. The Dzilam de Bravo system is one of the few scientifically documented cave system in the northern Yucatán coastline (YS)^13–15, 25^, contrasting the more than 1000 km^2^ of coastal cave passages that have been explored and documented along the northeastern edge of the Peninsula (QRS)^15,42,43^. Because of the distance between this and other coastal systems (> 200 km), previous work may not have considered the presence of *T. dzilamensis* in regions well outside of its previously known range or euryhaline tendencies, leading to identification errors. Furthermore, comparative salinity trials focused on ecophysiological response (measuring oxygen consumption) to salinity change in *Typhlatya* species supports a greater tolerance and lower stress response in *T. dzilamensis*, when compared to *T. pearsei*, and *T. mitchelli*^44^. Thus, recent (and pending) reports^43^ and this study, support *T. dzilamensis* as a salinity generalist able to traverse a vast range of salinities (2–37 psu), bringing caution to species identification solely based upon cave locality and/or sampling environment^45^.

Traditional taxonomy of *Typhlatya* species within the Yucatán Peninsula used rostrum length relative to the margin of the eyes, as a critical diagnostic character (Fig. 3)^24,25,46^. Rostrum length has been highlighted as a distinguishing character between *T. campecheae* and *T. pearsei*^25^, with both having a rostrum reaching beyond the margin of the eyes. In the original description^20^, *T. pearsei* was described as having a “short” rostrum. However, a redescription^24^ reported variable rostral length, but always extending beyond the first antennular segment of the peduncle (proximal base of antennule) somewhere between halfway through the second or just beyond the third antennular segment. This assessment of variability was based solely upon morphology and locality information, as specimens collected from re-designated type localities Gruta de Chac (YS), Cenote de Hoctún (YS), and Cenote Abejas (QRS)^24^ were reported to have a more extended rostrum than other locales. In contrast, *T. campecheae* is only known from the type locality, Grutas de Xtacumbilxunam (CS), and reported with a rostrum length reaching halfway or through the first antennular segment of the peduncle^24,25^. In the current study, the inclusion of individuals sampled from Campeche State (Grutas de Xtacumbilxunam and Cantemo), were genetically recovered within the *T. pearsei* clade (Fig. 4; SFig. 1) and indistinguishable from *T. pearsei* (SFig. 4A,B). *Typhlatya pearsei* has taxonomic priority^20^ over *T. campecheae*, potentially rendering the latter as a junior synonym. However, without access to fresh collections from the type locality of *T. pearsei* (Cenote Balankanché, YS), the taxonomic status of both species remains pending further investigation by colleagues (J. Mejia Arambula, Universidad Autónoma de Ciudad Juárez, pers. comm.). Variation in rostrum length within species is not uncommon and has also been reported in other members of Decapoda, such as members of the stygobitic genus *Troglocaris*^47^ and the deep-sea blue and red shrimp, *Aristeus antennatus*^48^*.* Therefore, combined morphological and molecular approaches, including material from the type locality of *T. pearsei* are imperative to clarify the identities of *T. pearsei* and *T. campecheae*, especially given their federally protected status in Mexico (see details below).

In addition to clarifying the identity of known species within the Yucatán Peninsula, two genetic lineages were newly revealed in our analyses, adding to the current taxonomic complexity (Fig. 4). *Typhlatya* sp. A was recovered as sister to *T. mitchelli* + *T. pearsei* and was not recovered among GenBank data (STable 4). Like *T. mitchelli*, *Typhlatya* sp. A has a short rostrum that does not extend beyond the margin of the eyes. However, the distal edge of the rostrum of *Typhlatya* sp. A appears thinner and less robust than that of *T. mitchelli* (SFig. 4C,E). *Typhlatya* sp. B was recovered as sister to *T. dzilamensis*, with one sequence previously misidentified as *T. mitchelli* (KX844712^22^). Unfortunately, voucher material for *Typhlatya* sp. B (16Tabanos01, 27Fbang06) was not available for morphological evaluation. However, previous photo-documentation^41^ of *Typhlatya* sp. B (i.e., *Typhlatya* sp. 1) showed the rostrum extending beyond the eyes, like *T. pearsei*, but not beyond the first antennular segment. Additional evaluation is pending to clarify these differences (J. Mejia Arambula, Universidad Autónoma de Ciudad Juárez, pers. comm.). It should be noted that these new lineages came to light post-genetic evaluation and based on few samples (n ≤ 7 each).

Another unexpected result was the syntopy among all five lineages within the meteoric lens (low salinity waters) of the OBH system (Fig. 5). Other caves also harbored at least two species (i.e., Actun Ha, Hoctún, Sistema Paamul). Thus, caution should be applied to species identification based upon morphology and assumed salinity preferences alone.

### Salinity preferences of anchialine Atyidae

Atyidae is a diverse, globally distributed family of predominantly freshwater shrimp species that includes multiple independent clades of stygobitic taxa, and so far one clade identified as anchialine^31,49^. To date, atyid species have not been reported from open ocean environments^31,49^. Association of anchialine atyids (*Halocaridinides* + *Halocaridina* + TST complex^22,31,46^) with saline waters (> 5 psu) is dominant across multiple lineages within this clade, contrasting only three transition to low salinity. While the ancestral reconstruction of salinity preferences was unresolved at deep nodes, the ancestor of the Yucatán + Cuba clade was unambiguously low salinity (< 5 psu). Except for the euryhaline *T. dzilamensis*, all remaining descendants within this clade inhabit the meteoric lens, suggesting that the *T. dzilamensis* lineage potentially transitioned from low to high salinity tolerant as it expanded across coastal cave systems. The Cuban *Typhlatya garciai* was originally reported as a freshwater species^50^, and later recorded from brackish/saline cave waters of Caicos Islands^51^. Representatives of *T. garciai* from both locations were not recovered as monophyletic (needing further taxonomic and phylogenetic evaluation), nor did its inclusion change the ancestral condition of the Yucatán + Cuba clade (SFig. 6).

The evolution of salinity preference (and tolerance) within anchialine fauna was likely influenced by historic sea-level change and proximity of cave systems to coastlines, where the marine influence is the greatest (Fig. 5). The hydrology of underlying aquifers has undergone drastic change (e.g., fresh ↔ anchialine ↔ marine) as evidenced by shifts in micro-paleontological benthic meiofauna over time^16,52–55^. Depending upon the salinity preference/tolerance of an anchialine species, sea-level fluctuation can likely result in significant changes to their respective range and distribution^55^. Subsequently, periods of isolation and expansion within a karst aquifer may have acted as drivers of diversification for anchialine fauna^55^.

The ability of *T. dzilamensis* to cross the salinity barrier offers a great advantage to expand ecological niches and access a broader range of carbon and energy sources than previously estimated, when compared to other stenohaline congeneric species of the Yucatán^44^. *Typhlatya dzilamensis* was observed above and below the halocline within OBH (QRS) and Crustacea (QRS), and syntopic, within a few kilometers, with remaining four species in the same cave system (e.g., OBH), and with *T. mitchelli* in Actun Ha (Tulum, QRS). *Typhlatya dzilamensis* also has an extensive range from at least the north/northwestern and eastern coastal margins (> 500 km); whereas *T. pearsei*, *T. mitchelli*, *Typhlatya* sp. A, and *Typhlatya* sp. B appear to be spatially restricted to the meteoric lens but span a great distance (~ 200 km) among inland (YS) and coastal caves (QRS) (Fig. 5A). Although *T. pearsei* was reportedly observed in fresh and marine layers of Cenote Crustacea^56^ extensive sampling within this system for this study suggests *T. dzilamensis* as the sole *Typhlatya* species that occurs there. Salinity profiles of Crustacea show a shallow meteoric lens (< 5 m depth), followed by two haloclines, each above a brackish (~ 8 psu) and marine (> 30 psu) layer, respectively (see Fig. 2). Field observations noted *Typhlatya* sp. A (i.e., *Typhlatya* sp. 2) may cross the halocline^41^. However, this may be indicative of a strategy of low salinity species to periodically search for prey items (or other food sources) and/or avoid predators (and divers) moving through the halocline while inhabiting coastal cave systems.

### Distribution and expansion within crevicular spaces

How anchialine fauna have achieved such disjunct distribution patterns despite inhabiting seemingly isolated environments remains a significant biogeographical question. *Typhlatya* species are interspersed throughout the Caribbean, West Indies, Bermuda, Yucatán Peninsula and in the West Atlantic^21,22^. However, without representatives of *Typhlatya* distributed throughout the Caribbean Islands (STable 8) in phylogenetic and biogeographic evaluations, the resolution of ancestral reconstructions and divergence dating estimations will remain limited and incomplete.

The Yucatán Peninsula is of particular interest, as it hosts the highest regional diversity of *Typhlatya* in the world. The Yucatán Peninsula and the Paleogene Arc (Cuba + Hispaniola) are hypothesized to have been connected via a land span in the Paleocene through Middle Eocene (until ~ 49 Mya) and then subsequently split after the formation of the Cayman Trough^57–59^. What has been termed the “Paleogene arc drift vicariance scenario” may explain the disjunct distribution patterns of modern-day taxa, such as those of certain fish (Cichlidae)^59^. Prior to this vicariant event, opportunities for ancestral expansion of *Typhlatya* within the meteoric lens of spelean corridors^60^ along the Paleogene arc would have been possible between Cuba^61^ and emerged portions of the Yucatán Peninsula^62^. *Typhlatya* species (and anchialine atyids) have not been reported outside of groundwater aquifers, yet open ocean dispersal to isolated oceanic islands (e.g., Bermuda) has been proposed^30,63^. In contrast, this study suggests that dispersal opportunities through established (e.g., deeper or ancient cave systems) or temporary tectonic (e.g., temporary land bridges) and/or volcanic connections (e.g., lava tubes) are plausible^30,59,64^ assuming the availability of suitable aquatic habitat^16,52–55^.

In this study, Yucatán + Cuba *Typhlatya* clade was estimated to have diverged and subsequently diversify in the Miocene or early Cenozoic (Table 1, STable 5), the former much later than the estimated split between Yucatán and Cuba. Although estimated ages of modern-day caves in the Yucatán date back to at least the Miocene within Carrillo Puerto Formation^65,66^. Divergence estimates to the Cretaceous (~ 75 Mya)^22^ was also proposed (Table 1; STables 5–6). During this time, however, the Yucatán platform was predominantly submerged, although global sea-level oscillations led to periods of partial submergence and emergence^52–54,62,67,68^. The formation of and position of the Paleogene Arc in proximity to Middle America did not occur until the late Cretaceous and early Paleocene^59^, respectively. Therefore, the possibility of shared ancestry between Yucatán and other Caribbean *Typhlatya* seems unlikely before this major event.

The precise timing and events leading to the diversification of modern Yucatán *Typhlatya* and transition to euryhaline in *T. dzilamensis* is difficult to discern without a fossil record, although present-day distributions appear to be greatly influenced by hydrogeology (Fig. 2), as seen in other taxa^69–71^. The genetic connectivity among Yucatán populations over great distances was explored within *T. mitchelli*^30^, where the analysis of the fast-evolving CYTB revealed low haplotypic diversity over distances of up to 235 km. Although haplotype diversity was not estimated in the current study, intra-clade pairwise genetic distances for *T. mitchelli*, *T. dzilamensis* and *Typhlatya* sp. B, were generally less than 2% for 16S and COI, respectively, thus supporting broad dispersal capabilities throughout the aquifer. Intra-clade pairwise genetic distances in *T. pearsei* and *Typhlatya* sp. A, were 2% and 5% (COI), respectively, between distant cave systems (e.g., *T. pearsei* (Odyssey, QRS) vs. *Typhlatya c.f. campecheae* (Cantemo, CS): 5.3%), potentially indicating geographic population structure. An optimal next step would be to assess population connectivity amongst all *Typhlatya* species in the Yucatan to elucidate dispersal capabilities.

### Implications for continuing integrative research

Biodiversity management and conservation of karst aquifers^72^ need more studies with an inter-disciplinary research framework via the collection of integrative data (e.g., phylogeny, morphology, DNA barcoding, hydrogeology/water quality, biogeochemistry, paleoecology, etc.)^73,74^. This study serves as a case study to provide guidance (and caution) for continuing research on poorly studied stygobitic biodiversity of the Yucatán aquifer.

The current findings bring forth the need for revision and re-evaluation of the conservation status of *Typhlatya* species within the Yucatán Peninsula. The Official Mexican Standards for Environmental Protection (NOM-059-SEMARNAT-2010)^23^ lists three of four known species: *T. campecheae* as federally endangered*,* and *T. mitchelli* and *T. pearsei* as federally threatened, while IUCN Red List classifies these species as stable and of least concern^75^. The results unambiguously showed that *T. campecheae,* recorded as endemic to Grutas de Xtacumbilxunam (CS), may not be distinct species from the *T. pearsei*. Further, our findings support the expansive distribution of *T. pearsei* within the meteoric lens from inland (YS and CS) to coastal (QRS) cave systems, with *T. mitchelli* having an overlapping range but not found syntopically in this study, except for the coastal Ox Bel Ha system (QRS); not recorded from CS caves (to date). *Typhlatya dzilamensis* is not federally listed and yet was here found to be distinct from all other species, representing the only euryhaline species with a habitat range restricted to coastal cave systems throughout the Peninsula. Thus, *T. dzilamensis* is an excellent candidate in need of evaluation, given its association with karst coastlines, which are particularly vulnerable to anthropogenic impacts and coastal development changes^72,76,77^. Lastly, the identification of two unknown species, which were ‘rare’ among our newly sequenced samples and in previously published data, warrants further study to better understand their distributions and population genetic structure. Pending species descriptions, they are likely candidates for elevated conservation status.

The presence of *Typhlatya* within inland and coastal caves throughout the Peninsula, and their strict affinity to particular salinities and hydrogeological constraints, identifies member species as potential models to assess groundwater health. Given continuing sea-level rise^52–54^, temporal high energy meteorological events (e.g., hurricanes)^78^, and other environmental impacts^72^ prevalent in the Yucatán region, *Typhlatya* species can serve as biological indicators^79,80^ and/or indicators of ecological resilience^81^ to environmental change. The phylogeny presented here provides a robust evolutionary hypothesis for conducting comparative studies among closely related species to understand adaptation and key functional roles within these ecosystems^74^. The presence of a salinity generalist within subterranean estuaries, like *T. dzilamensis,* provides an excellent opportunity to evaluate the functional role of this adaptation in carbon-energy transfer from the meteoric lens (where organic matter is abundant) to the extremely oligotrophic saline groundwater^11^.

The aforementioned would be challenging without discrete species clarity^82^. Therefore, the *Typhlatya* research presented here can serve as a baseline for integrative and multi-disciplinary investigations with local experts and researchers, to provide robust scientific evidence to support policy, conservation efforts and biodiversity assessments of the extensive karst aquifer ecosystems within the Yucatán Peninsula.

## Methods

### Taxon sampling

Sixty-three individuals within the genus *Typhlatya* were collected during several field expeditions to caves throughout the Yucatán Peninsula between 2013 and 2019 (see Fig. 5A, B, STables 3, 4). Efforts were taken to collect representatives from type localities to address contentious identifications, namely *T. dzilamensis* from Cenotes Buya-Uno, Cervera, and Dzilamway (Dzilam de Bravo, YS) and *T. campecheae* from Grutas de Xtacumbilxunam (CS). Samples from the type locality (Grutas de Balankanche) of *T. pearsei* were not available for this study due to its status as a protected archaeological site and tourist attraction. Initial morphological identification of specimens (except from specimens sampled below, OBH) followed Álvarez et al*.*^25^. At the time of finalizing this study, access to type material located at the Smithsonian Institution National Museum of Natural History was not possible due to COVID-19 restriction access to the research collections.

Collections of *Typhlatya* were also conducted from four cenotes (Na’ach Wennen Ha (Tabanos), Odyssey, Jailhouse, Bang) within the OBH cave system (QRS) along a geographic transect from the Caribbean coast (Fig. 5), as first described by Benitez et al.^43^. Specimens were also collected from Cenote Crustacea (QRS) and the Dzilam de Bravo (type locality of *T. dzilamensis*). Specimens from these sites were collected “blindly” (i.e., without identifying to species prior to preservation or sequencing) only making note of the environmental conditions (i.e., salinity, temperature, depth) within cave sections of specimen collection. Individuals were collected by cave divers using sampling bottles. Sampling depth and salinity were measured when possible, using either YSI XLM-600, EXO-02, Hydrolab DSS, or SonTek CastAway CTD devices. Additional profiles (see Fig. 2) were taken with a Hydrolab DataSonde5X within Xtabay (Tulum, QRS; 29 October 2017), Noh-Mozon (Pixya, YS; 15 March 2019), Tza Itza (23 August 2017), Crustacea (Puerto Morelos, QRS; 13 March 2019), and Cervera (Dzilam, YS; 02 December 2016).

Salinity concentrations were classified as followed: Low salinity (0.5–4.9 psu), oligohaline meteoric lens and generally above primary halocline, and High salinity (5–37 psu), mesohaline to polyhaline/euhaline groundwater and generally below the primary halocline; we recognize that these are not discrete and some species may exhibit overlap in discrete salinity designations (e.g., *T. dzilamensis*). These designations are based on collection of specimens for this study and simplified from previous salinity classifications^83^. The primary halocline is recognized as the mixing zone below the meteoric lens, where salinity levels can sharply increase to mesohaline (e.g., ~ 10 psu, Crustacea, Puerto Morelos, QRS) to euhaline (> 30 psu; e.g., Xtabay, QRS); see Fig. 2. Salinities above 5 psu were not observed within the meteoric lens of collection sites in this study. Salinities below the halocline were variable (and may include multiple haloclines), ranging from mesohaline to saline (Fig. 2)^83^.

Specimens with known salinities are indicated in Fig. 4B and STable 3. Within six hours of collection from the OBH transect, specimens were wrapped and stored at 0 °C in prebaked (450 °C for 4 h) aluminum foil. The specimens were transported frozen on dry ice, and then stored in the laboratory at − 20 °C. While frozen, select specimens were subsampled (abdomen) for molecular analysis and transferred to 95–100% ethanol, with the remaining anterior portion used for stable isotope and fatty acid analyses^11^. Specimens collected from Tza Itza, Nohmozon, Ponderosa via Xtabay and Kankeriche were prepared for carbon tracing^43^ and subsampled (pleopods) for genetic identification. Other collected material was fixed in ethanol (90–100%) immediately, or frozen with liquid Nitrogen or dry ice, and stored at − 20 °C or − 80 °C until ready for DNA extraction. Morphological and molecular vouchers are deposited at the Collection of Crustacea, UNAM-Sisal, Yucatán, MX (STable 4).

### GenBank selection, DNA extraction

Available mitochondrial and nuclear gene sequences for *Typhlatya* species (*T. pearsei*, *T. mitchelli*, *T. dzilamensis*, and *Typhlatya* sp.) from the Yucatán Peninsula were downloaded from GenBank (n = 112; STable 4). This dataset included gene fragments of mitochondrial 16S rRNA, COI (two partial fragments) and CYTB, and nuclear H3, 18S rRNA, and 28S rRNA. Of all genes, 16S rRNA was one of the most consistently utilized gene across separate studies and therefore represents the broadest sampling used for the phylogenetic evaluations presented here. Multi-gene analyses included select individuals as clade representatives. Individual genes trees of COI, CYTB, H3, 18S rRNA, and 28S rRNA are provided within the supplemental (Figs. 1–5).

Tissue was removed from 63 individuals and DNA was extracted following standard protocols of either ethanol precipitation or the Qiagen Dneasy Tissue and Blood kit. Genes 16S rRNA, COI, CYTB, H3, 18S rRNA, and 28S rRNA were amplified using primers outlined in STable 9. PCR mixtures consisted of DNA template (1–2 μL), forward primer (1 μL), reverse primer (1 μL), water (9.5 μL), and GoTaq^®^ Green Mastermix (12.5 μL). PCR temperature profiles are specific to each primer and were run using the BIORAD T100 Thermocycler. Successful amplifications was observed via gel electrophoresis and purified with ExoSAP-IT PCR Product Cleanup kit. Cleaned PCR products were then sent for sequencing to the Texas A&M Corpus Christi Genomics Core Lab. Geneious Prime 2020 v.2.3–2021 v.0.1^84^ was used to examine, clean, and assemble raw sequence data. Protein-coding genes were checked for the presence of stop-codons and pseudogenes and were checked for contamination via NCBI Blast. Newly generated gene data (16S = 40; COI = 8; CYTB = 11; 18S = 11; 28S = 13; H3 = 14) were subsequently submitted to GenBank (STable 4). Additional COI sequences (n = 21) were utilized from the Chavez-Diaz undergraduate thesis^41^ and submitted to GenBank (STable 4).

### Phylogenetic analyses

The 16S rRNA dataset was compiled from both GenBank (n = 34) and newly generated data (n = 41). Sixteen of the forty-one newly sequenced individuals were used for the multi-gene concatenated analyses (16S rRNA, COI, CYTB, H3, 18S rRNA, 28S rRNA; n = 5514 bp). Individuals were selected based upon sampling locality, species identification, and sequencing success of at least three of the targeted six genes. Gene alignments were concatenated using SequenceMatrix^85^. Sequence data for each gene were aligned via MAFFT^86^ local iterative (18S, COI, CYTB, H3) and global iterative method (16S, 28S). Phylogenetic analyses using both Maximum Likelihood (ML) and Bayesian Inference (BI) were conducted on XSEDE within the CIPRES Science Gateway^87^. *Typhlatya consobrina* Botoşăneanu & Holthuis, 1970, *Typhlatya garciai* Chace, 1942, and *Typhlatya taina* Estrada & Gómez, 1987 were selected as outgroup taxa based upon previous phylogenetic assessments^22^. Different individuals from the same outgroup species (*T. taina* and *T. consobrina*) were concatenated together to provide the largest available dataset for comparison (see STable 4). Optimal partitioning schemes and substitution models were selected within IQ-TREE^88^ based upon BIC (see STable 10). Maximum likelihood analyses were conducted within IQ-TREE and node support quantified via ultrafast bootstrapping with 1000 replicates. Parameters -nstop 500 and -pers 0.2 were implemented to reduce the likelihood of settling upon local optima. Bayesian analyses were conducted using MrBayes v.3.2.7a^89^ with 30 million generations, a burn-in of 10 million, sample frequency every 1,000 generations, and with two runs and four chains. Tracer^90^ v. 1.6.0 was used to confirm convergence of individual chains and examine effective sample size (ESS > 200). Analyses were visualized using FigTree^91^ version 1.4.4 (Fig. 4).

### Divergence dating

Divergence dating of nodes was performed using BEAST2^92^ v.2.6.3 based on multi-gene (16S rRNA, COI, CYTB, 18S rRNA, 28S rRNA, H3) datasets. The strict and uncorrelated log-normal clock models were used to explore the estimation of node ages. Divergence dating analyses used gene partition schemes and substitution models as selected by PartitionFinder via IQ-TREE (exploring with and without the 3rd codon position of protein coding genes; STable 7). Estimation of Bayes Factors and marginal likelihoods for a combination of partition schemes and clock models were conducted with path-sampling analyses in BEAST2 to select the best fit model for subsequent analyses (STable 7). Site models for each partition used empirical gamma shape, proportion of invariable sites and substitution rates as estimated by PartitionFinder in IQTree. Partition substitution rates were not estimated due to the complex partitioning scheme. Two independent runs for each analysis were performed for 100 million generations (Anchialine Atyidae) or 50 million generations (Late Cenozoic Clade), and sampled every 1000 generations. Outputs from each run (.log and .trees files) were combined using LogCombiner^92^ v.2.6.3, excluding the initial 10% of the burn-in, checked for convergence using Tracer^90^ v.1.4.4 and trees, mean values and confidence intervals were summarized using TreeAnnotator^92^ v.2.6.3. Analyses were conducted based on expanded taxon sampling beyond the Yucatán clade, detailed in STable 11; also used for stochastic mapping analyses.

This Anchialine Atyidae clade included members of the *Typhlatya/Stygiocaris/Typhlopatsa* (TST) complex, following Jurado-Rivera et al.^22^, as well as *Typhlatya galapagensis* (Islas Isabella/Santa Cruz), *Halocaridina rubra* (Hawaiian Islands) and *Halocaridinides fowleri/H. trigonophthalma* (concatenated) as outgroup taxa. Trees were rooted with *H. fowleri/H. trigonophthalma*. The timing of biogeographic events^21^ and/or approximate node age of the TST-complex previously estimated from fossil calibrations^22^ were used to calibrate molecular clocks (summarized in STable 7): (a) isolation of *T. galapagensis* divergent populations (8.0% pairwise distance) on Isabella and Santa Cruz Islands, with the ancestor not older than the Coco Ridge; 5–14 Mya; (b) isolation of *Stygiocaris lancifera* Holthuis, 1960 and *Stygiocaris stylifera* Holthuis, 1960 ancestor post emergence of the Cape Range anticline (Australia); 7–10 Mya and (c) isolation of the *T. consobrina* + *T. taina* ancestor post Havana–Matanzas Channel closure (Cuba)*;* 5–6 Mya. Additionally, the approximate age for the diversification of *Halocaridina rubra* Hawaiian complex^64^ was also included based on minimum ages of their presence on the islands (0.5–5 Mya)^93^ but does not estimate the age of the origin of the species, which is likely much older. Inclusion of divergent crustacean fossils used in previous work^22^ was not evaluated here. Instead, secondary date estimates (fossil calibrated) for the TST complex^22^ were used as a prior (i.e., ~ 149–118 Mya) for this clade.

### Stochastic mapping of salinity preference

Ecological transition of salinity preference within anchialine Atyidae was reconstructed via stochastic mapping over the ultrametric tree from divergence dating analyses (above) and used phytools^94^, ape^95^, and geiger^96^ packages within R Studio^97^. Salinity data was compiled from the literature and observations from the present study (see STables 3, 11). Due to the absence of exact salinity values within a majority of the literature (often being described as “freshwater” or “saltwater” habitats), the salinity trait was generalized to discrete data with two states: (1) low salinity and (2) high salinity. In the case of missing ecological data from GenBank material, salinity characters were assigned to each species based upon historical descriptions of the cave locales and previous sampling. To determine whether salinity states have transitioned at equal or unequal rates, both models were compared using the AIC.

Five hundred simulations (50,000 MCMC generations) were generated for stochastic character mapping using SIMMAP^98^, assuming equal rates and an unequal estimation at the root (0.75 likelihood of low salinity ancestry based upon the previous phylogenetic hypotheses of Atyidae^31^). These simulations were visually condensed to illustrate the probability density of each discrete character at internal nodes (Fig. 6). A secondary reconstruction was generated using the “Cenozoic” clade assuming equal rates and an equal estimation at the root (based upon the previous analysis) with 500 simulations at 50,000 MCMC generations (SFig. 6).

### Map construction

The distribution of 109 individuals of *Typhlatya* within the Yucatán Peninsula was visualized using software program QGIS v.3.1^99^ of which, 62 locality records were obtained from previous studies^21,22,29–34,41^ and 42 records are newly generated from this study. Species identities were assigned from the single-gene phylogenies of the present study (above). Vector map data was obtained from Natural Earth (www.naturalearthdata.com). Cenotes of the type locality of *T. dzilamensis* (Dzilam de Bravo, Cervera) were treated as one locality.

### Species delimitation

To assess the number of phylogenetic entities within this dataset, species delimitation methods were performed via Generalized Mixed Yule Coalescent (GMYC)^100^ and the Poisson Tree Process (PTP)^101^ Single genes that contained all five hypothesized lineages (16S, COI, CYTB) were selected, with duplicate sequences removed from each alignment and the species *Halocaridina rubra* (KF437502) implemented as the outgroup. In each analysis, single genes were assessed (COI and CYTB partitioned by codon) as well as a concatenated three-gene analyses (partitioned by gene). For GMYC analyses, ultrametric trees were constructed using BEAST2 v. 2.6.6 via the Cipres Science Gateway^87^. Input files were generated within BEAUti v.2.6.6 assuming a strict clock, chain length of 30,000,000, with site models selected using bModelTest^102^. Both the Yule and Coalescent with constant population models were tested per single-gene and concatenated analyses. Tree Annotator v. 2.6.4 was used to generate a maximum clade credibility tree with 10 percent burnin for each analysis and Tracer^90^ v.1.6.0 was used to assess convergence (posterior, likelihood, and prior ESS > 200). GMYC analyses were conducted using the SPLIT^103^ package of Rstudio^97^ v.1.3.1093. For PTP and Bayesian PTP (bPTP) analyses, trees were constructed via IQ-TREE^88^ within the Cipres Science Gateway^87^. Both PTP and bPTP were ran within the Exelixis web server (https://species.h-its.org/ptp/) at 100,000 MCMC generations with the outgroup *Halocaridina rubra* excluded.

## Supplementary Information

Supplementary Information.

## Data Availability

Newly obtained sequences were deposited in GenBank Repositories under accession numbers listed in STables 4 and 11. All data, including phylogenetic trees and R-scripts used throughout this study are publicly available on the Open Science Framework (OSF) repository (https://osf.io/fve6q/?view_only=0a1bf5879bc0432ba356d8a8c2dc0fbd).
