# Supplementary material for: An integrative re-evaluation of Typhlatya shrimp within the karst aquifer of the Yucatán Peninsula, Mexico
Source: Sci Rep. 2022 Mar 29;12:5302. doi: 10.1038/s41598-022-08779-9 (PMC8961266; doi:10.1038/s41598-022-08779-9)
Supplement: Supplementary file 1 — Supplementary Information. [file 41598_2022_8779_MOESM1_ESM.pdf]

An integrative re-evaluation of *Typhlatya* shrimp within the karst aquifer of the Yucatán Peninsula, Mexico.

Lauren Ballou, David Brankovits, Efraín M. Chávez Solís, Brett C. Gonzalez, José M. Chávez Díaz, Shari Rohret, Alexa Salinas, Arielle Liu, Nuno Simões, Fernando Alvarez, Maria Pia Miglietta, Thomas M. Iliffe, and Elizabeth Borda

Supplementary Information

i. SFigures 1–3. Single gene phylogenetic hypothesis of *Typhlatya* spp. from the Yucatán Peninsula.
ii. SFigure 4. Morphological comparison of *Typhlatya* spp. within the Yucatán Peninsula.
iii. Provisional Key to *Typhlatya* species of the Yucatán Peninsula.
iv. SFigure 5. Chronogram of alternate divergence dating estimations for Anchialine Atyidae.
v. SFigure 6. Alternative stochastic mapping of salinity trait evolution of *Late Cenozoic* Clade, including *Typhlatya garciai* (Caicos Is).
vi. STable 1. Species delimitation analyses results.
vii. STable 2. Uncorrected pairwise (p) distances among Yucatán *Typhlatya* species.
viii. STable 3. Salinity and locality data for *Typhlatya* representatives.
ix. STable 4. Taxon sampling and GenBank data information for *Typhlatya* representatives.
x. STable 5. Divergence dating results and summary.
xi. STable 6. Alternative node age estimates.
xii. STable 7. Model selection process for BEAST analyses.
xiii. STable 8. List of *Typhlatya* diversity and distribution.

xiv. STable 9. Primers and temperature profiles for PCR reactions for six genes.
xv. STable 10. Model selection per gene for phylogenetic analyses.
xvi. STable 11. Taxon sampling and gene data information for anchialine Atyidae used for divergence dating and stochastic mapping analyses

i. **SFigures 1–3.**

SFigures 1–3. Single gene phylogenetic hypothesis of *Typhlatya* spp. from the Yucatán Peninsula, Bayesian phenogram shown. SFigure 1. COI; SFigure 2. CYTB and H3; and SFigure 3. 18S rRNA and 28S rRNA. Support values indicated at nodes above branches: Bootstrap/Bayesian Posterior Probability. Colors represent species identity resulting from this study: *Typhlatya pearsei* = yellow, *Typhlatya mitchelli* = red, *Typhlatya dzilamensis* = blue, *Typhlatya* sp. A = purple, *Typhlatya* sp. B = orange. Sequences from specimens from type localities indicate by colored stars (A, B), green star = *T. campecheae*, Grutas de Xtacumbilxunam; blue star = *T. dzilamensis,* Dzilam de Bravo.

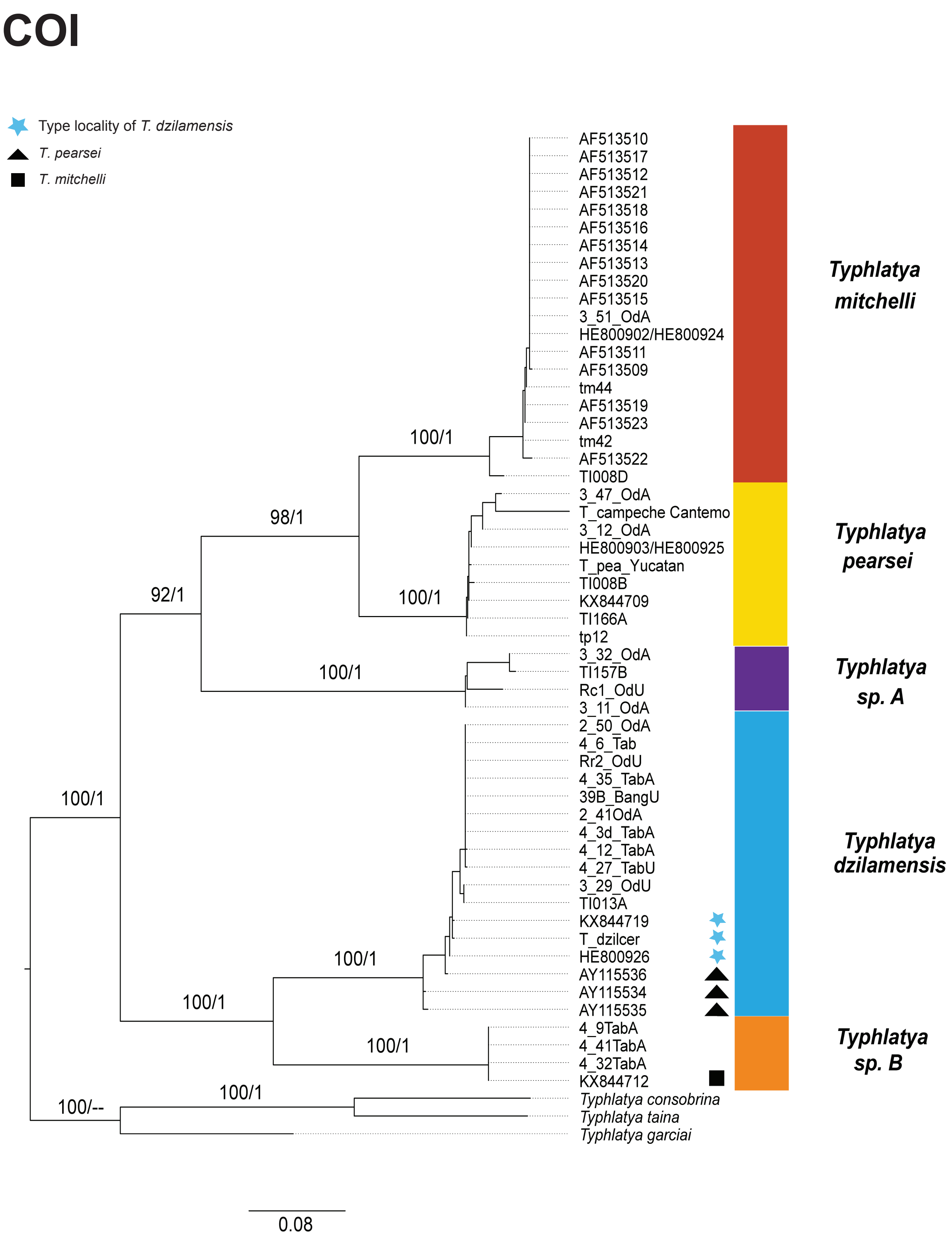
COI
Type locality of T. dzilamensis
T. pearsei
T. mitchelli
AF513510
AF513517
AF513512
AF513521
AF513518
AF513516
AF513514
AF513513
AF513520
AF513515
3_51_OdA
HE800902/HE800924
AF513511
AF513509
tm44
AF513519
AF513523
tm42
AF513522
TI008D
3_47_OdA
T_campeche Cantemo
3_12_OdA
HE800903/HE800925
T_pea_Yucatan
TI008B
KX844709
TI166A
tp12
3_32_OdA
TI157B
Rc1_OdU
3_11_OdA
2_50_OdA
4_6_Tab
Rr2_OdU
4_35_TabA
39B_BangU
2_41OdA
4_3d_TabA
4_12_TabA
4_27_TabU
3_29_OdU
TI013A
KX844719
T_dzilcer
HE800926
AY115536
AY115534
AY115535
4_9TabA
4_41TabA
4_32TabA
KX844712
Typhlatya consobrina
Typhlatya taina
Typhlatya garciai
Typhlatya mitchelli
Typhlatya pearsei
Typhlatya sp. A
Typhlatya dzilamensis
Typhlatya sp. B
100/1
98/1
92/1
100/1
100/1
100/1
100/1
100/1
100/1
100/1
100/--
0.08

CYTB

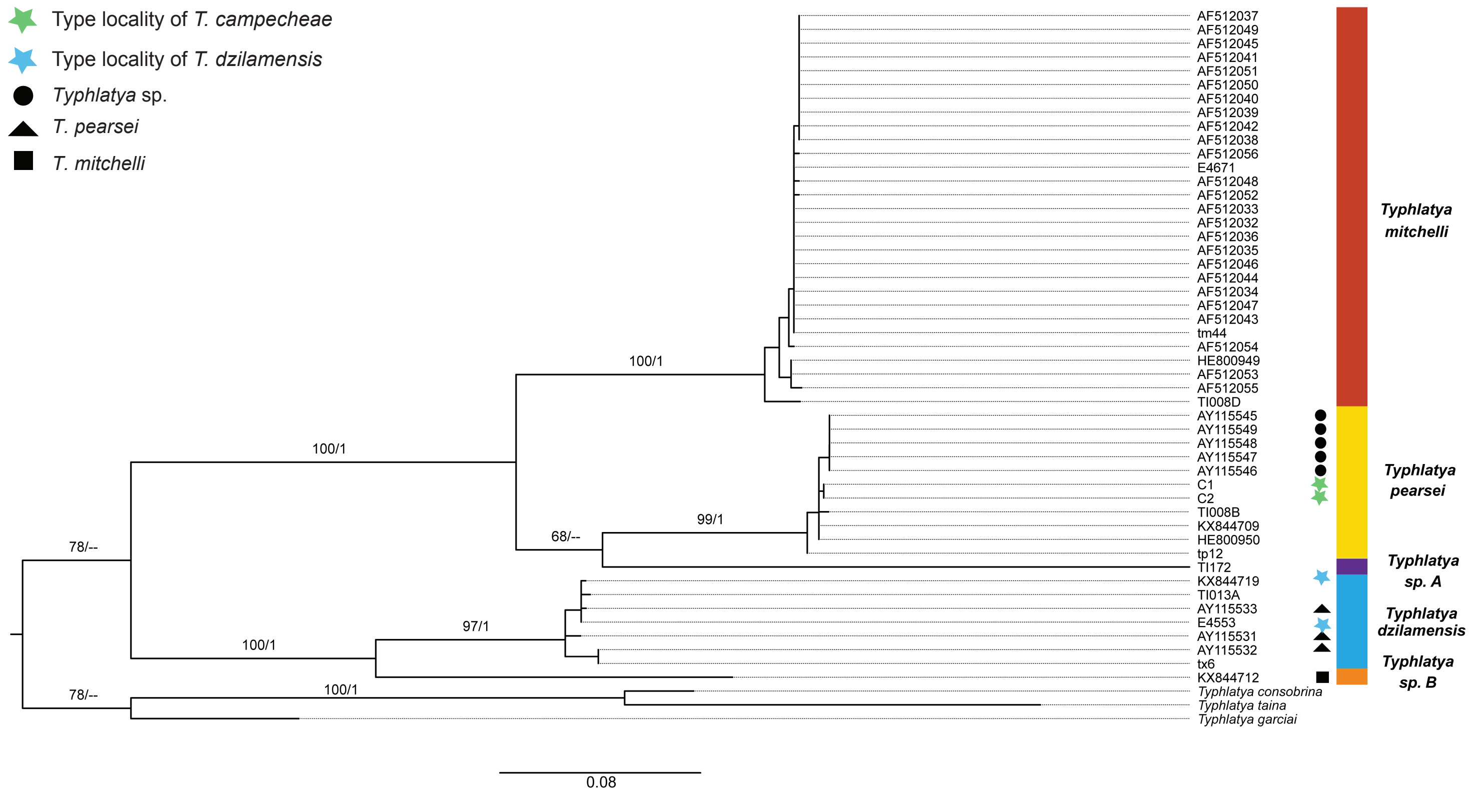

Type locality of T. campecheae
Type locality of T. dzilamensis
Typhlatya sp.
T. pearsei
T. mitchelli
AF512037
AF512049
AF512045
AF512041
AF512051
AF512050
AF512040
AF512039
AF512042
AF512038
AF512056
E4671
AF512048
AF512052
AF512033
AF512032
AF512036
AF512035
AF512046
AF512044
AF512034
AF512047
AF512043
tm44
AF512054
HE800949
AF512053
AF512055
TI008D
AY115545
AY115549
AY115548
AY115547
AY115546
C1
C2
TI008B
KX844709
HE800950
tp12
TI172
KX844719
TI013A
AY115533
E4553
AY115531
AY115532
tx6
KX844712
Typhlatya consobrina
Typhlatya taina
Typhlatya garciai
100/1
100/1
68/--
99/1
78/--
97/1
100/1
78/--
100/1
0.08
Typhlatya mitchelli
Typhlatya pearsei
Typhlatya sp. A
Typhlatya dzilamensis
Typhlatya sp. B

H3

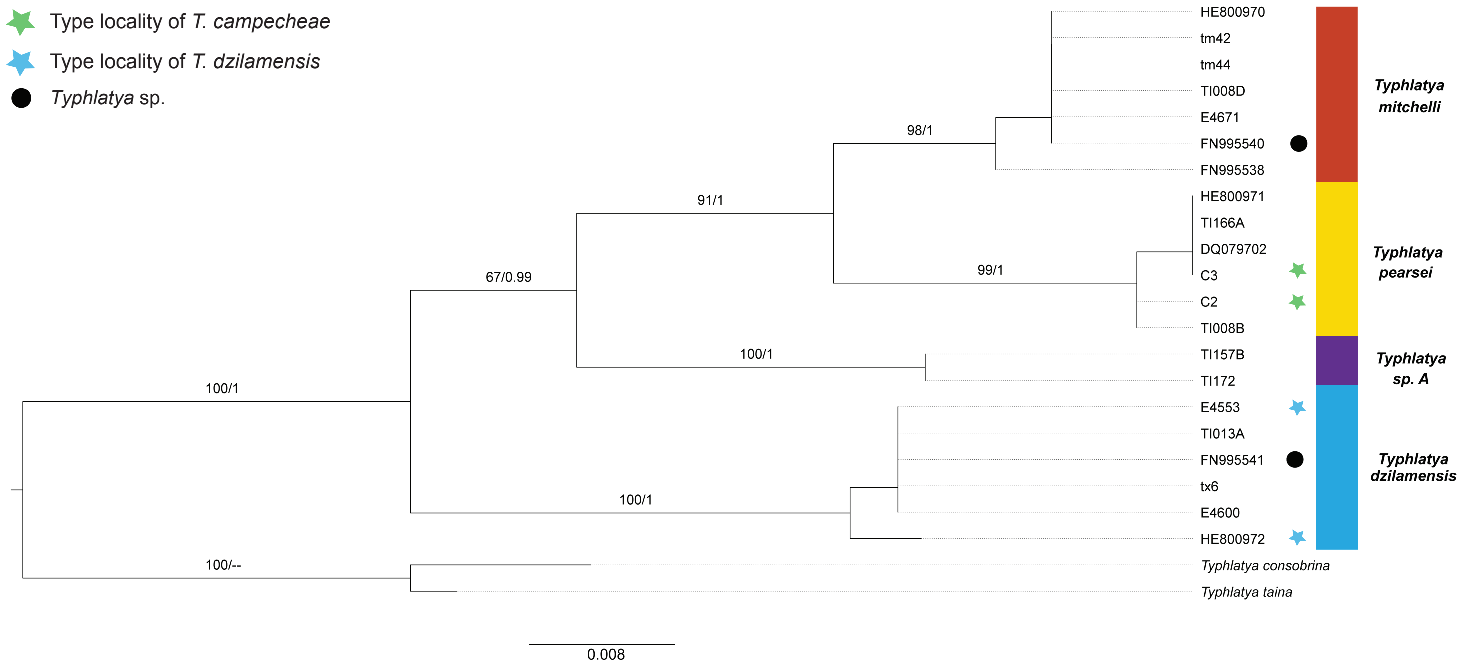

Type locality of T. campecheae
Type locality of T. dzilamensis
Typhlatya sp.
HE800970
tm42
tm44
TI008D
E4671
FN995540
FN995538
HE800971
TI166A
DQ079702
C3
C2
TI008B
TI157B
TI172
E4553
TI013A
FN995541
tx6
E4600
HE800972
Typhlatya consobrina
Typhlatya taina
98/1
91/1
99/1
67/0.99
100/1
100/1
100/1
100/--
0.008
Typhlatya mitchelli
Typhlatya pearsei
Typhlatya sp. A
Typhlatya dzilamensis

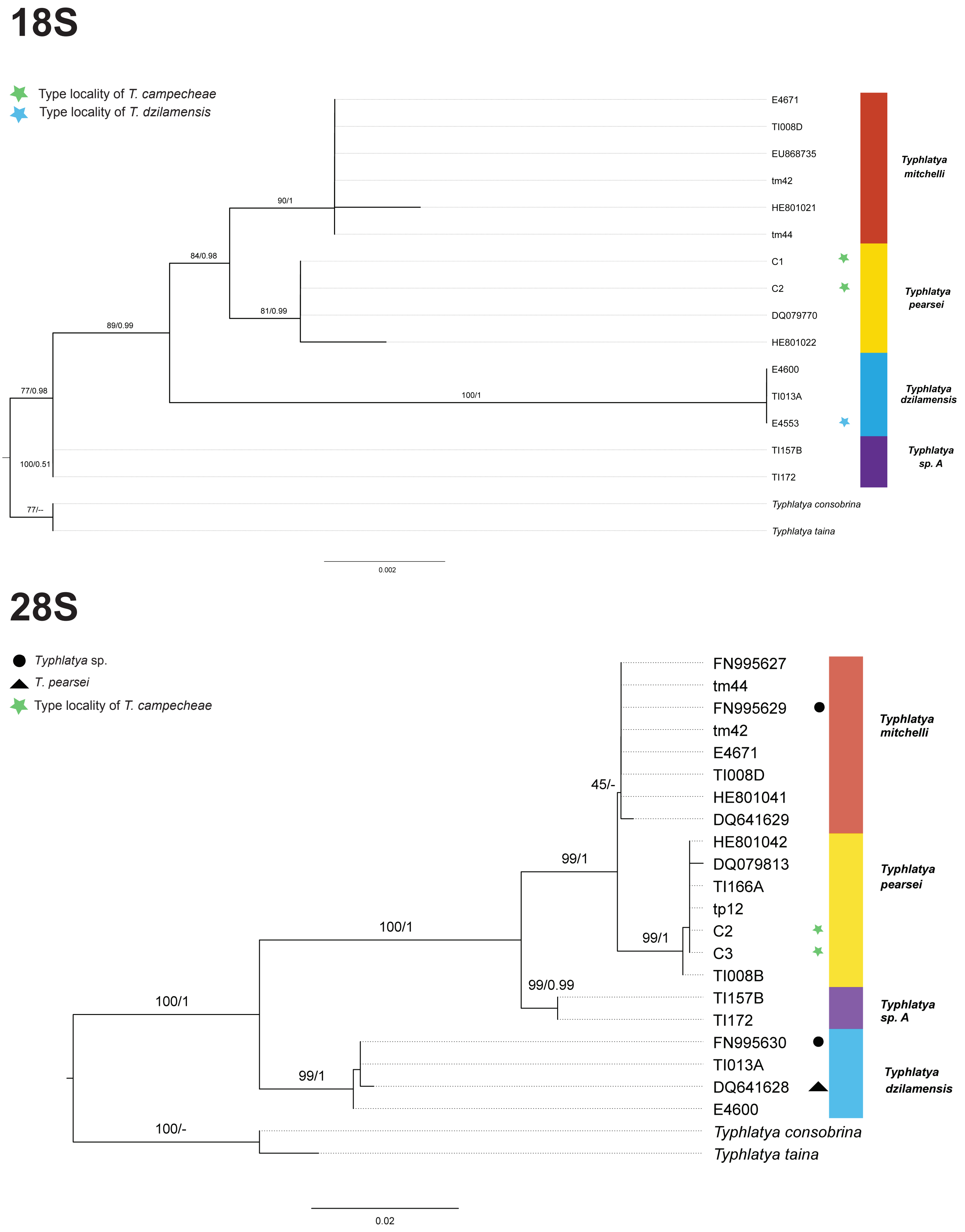
18S
Type locality of T. campecheae
Type locality of T. dzilamensis
E4671
TI008D
EU868735
tm42
HE801021
tm44
C1
C2
DQ079770
HE801022
E4600
TI013A
E4553
TI157B
TI172
Typhlatya consobrina
Typhlatya taina
90/1
84/0.98
81/0.99
89/0.99
100/1
77/0.98
100/0.51
77/--
0.002
Typhlatya mitchelli
Typhlatya pearsei
Typhlatya dzilamensis
Typhlatya sp. A
28S
Typhlatya sp.
T. pearsei
Type locality of T. campecheae
FN995627
tm44
FN995629
tm42
E4671
TI008D
HE801041
DQ641629
HE801042
DQ079813
TI166A
tp12
C2
C3
TI008B
TI157B
TI172
FN995630
TI013A
DQ641628
E4600
Typhlatya consobrina
Typhlatya taina
45/-
99/1
100/1
99/1
99/0.99
100/1
99/1
100/-
0.02
Typhlatya mitchelli
Typhlatya pearsei
Typhlatya sp. A
Typhlatya dzilamensis

ii. SFigure 4.

SFigure 4. Morphological comparison of *Typhlatya* spp. within the Yucatán Peninsula. A. *Typhlatya pearsei* = *Typhlatya campecheae* (C1*/C2*, Grutas de Xtacumbilxunam); B. *T. pearsei* (TI008B*, Systema Paamul; TI166A*/TI166B, Sabak Ha); C. *T. mitchelli* (TI008D*/TI008E, Systema Paamul; TP012*, Noh-Mozon); D. *T. dzilamensis* (TI005, TI015, TI013A*, Carwash) and *Typhlatya* sp. A (TI157A/TI157B*, Nayah; TI172*, Kankirixche). *Typhlatya* sp. B was not available for morphological evaluation. a, antennular peduncle; i/ii/iii, segments of antennular peduncle; e, eyes; r, rostrum. See also Provisional Key to Yucatán *Typhlatya* in the text. *Genetically identified, Figure 3.

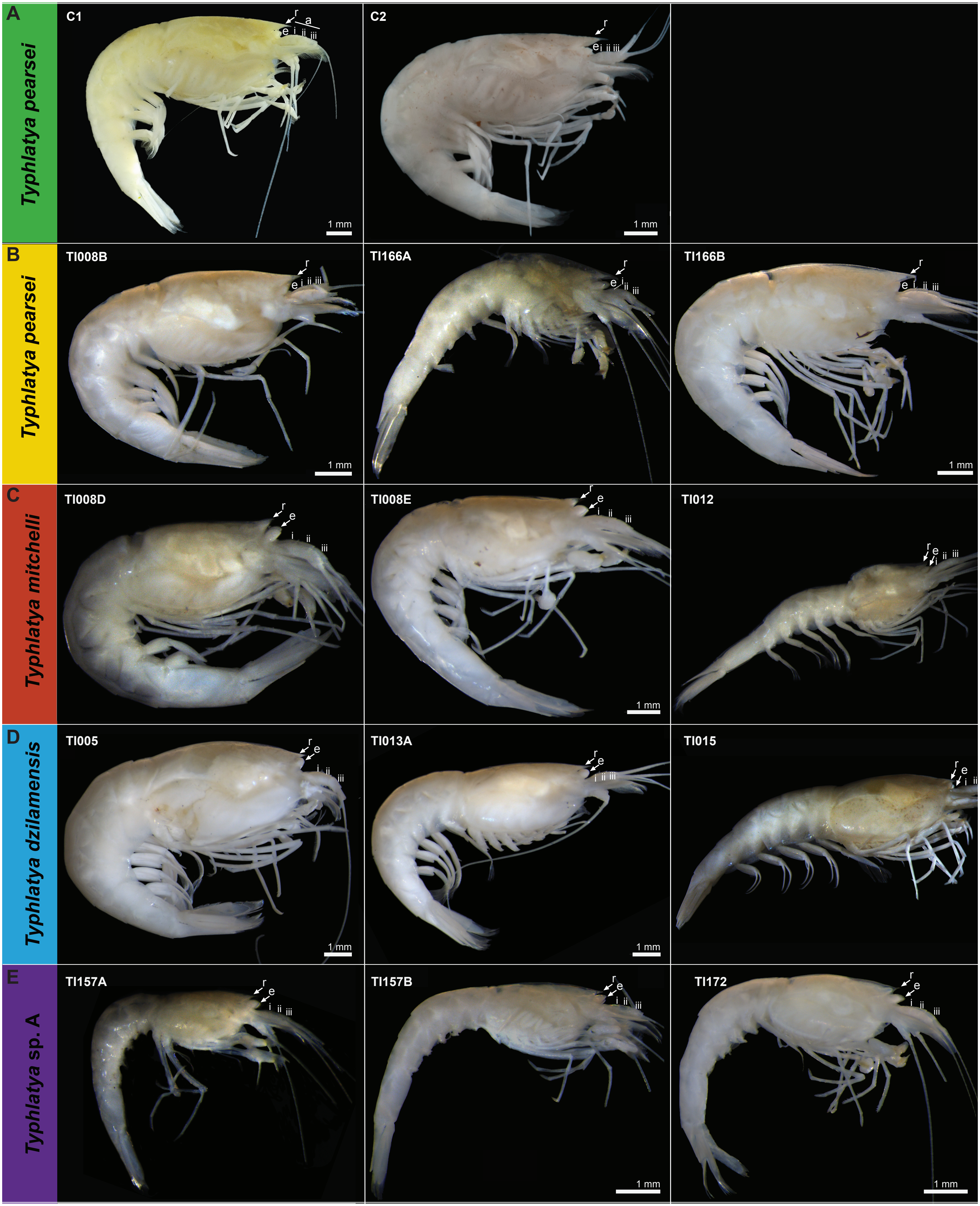
A
Typhlatya pearsei
C1
C2
B
Typhlatya pearsei
TI008B
TI166A
TI166B
C
Typhlatya mitchelli
TI008D
TI008E
TI012
D
Typhlatya dzilamensis
TI005
TI013A
TI015
E
Typhlatya sp. A
TI157A
TI157B
TI172
r
e
a
i
ii
iii
1 mm

iii. Provisional Key to *Typhlatya* species of the Yucatán Peninsula

1. Rostrum extending to and beyond the first antennular segment ……………… *Typhlatya pearsei*

-- Rostrum not extending to the end or beyond the first antennular segment ……………….…… 2

2. Rostrum shorter than eyestalk……………………………………………………..…….3

--Rostrum extends to eyestalk or just beyond, with broad carapace…………*Typhlatya dzilamensis*

3. Rostrum margin robust and thick with distinct upward curvature……………*Typhlatya mitchelli*

-- Thin and flat rostrum margin without upward curvature………………………...*Typhlatya* sp. A

iv. **SFigure 5.**

SFigure 5. Chronogram of altering divergence dating estimations [in brackets; Mya, 95% high posterior density limits (HPD) as confidence intervals] for Anchialine Atyidae. Chronogram based on analyses with relaxed, log normal clock, Yule model, 5 gene partitions and 5 independent clock models. Calibration priors (stars at nodes) based on geological events and secondary fossil calibrated estimation for the TST complex as described in STables 5-7.

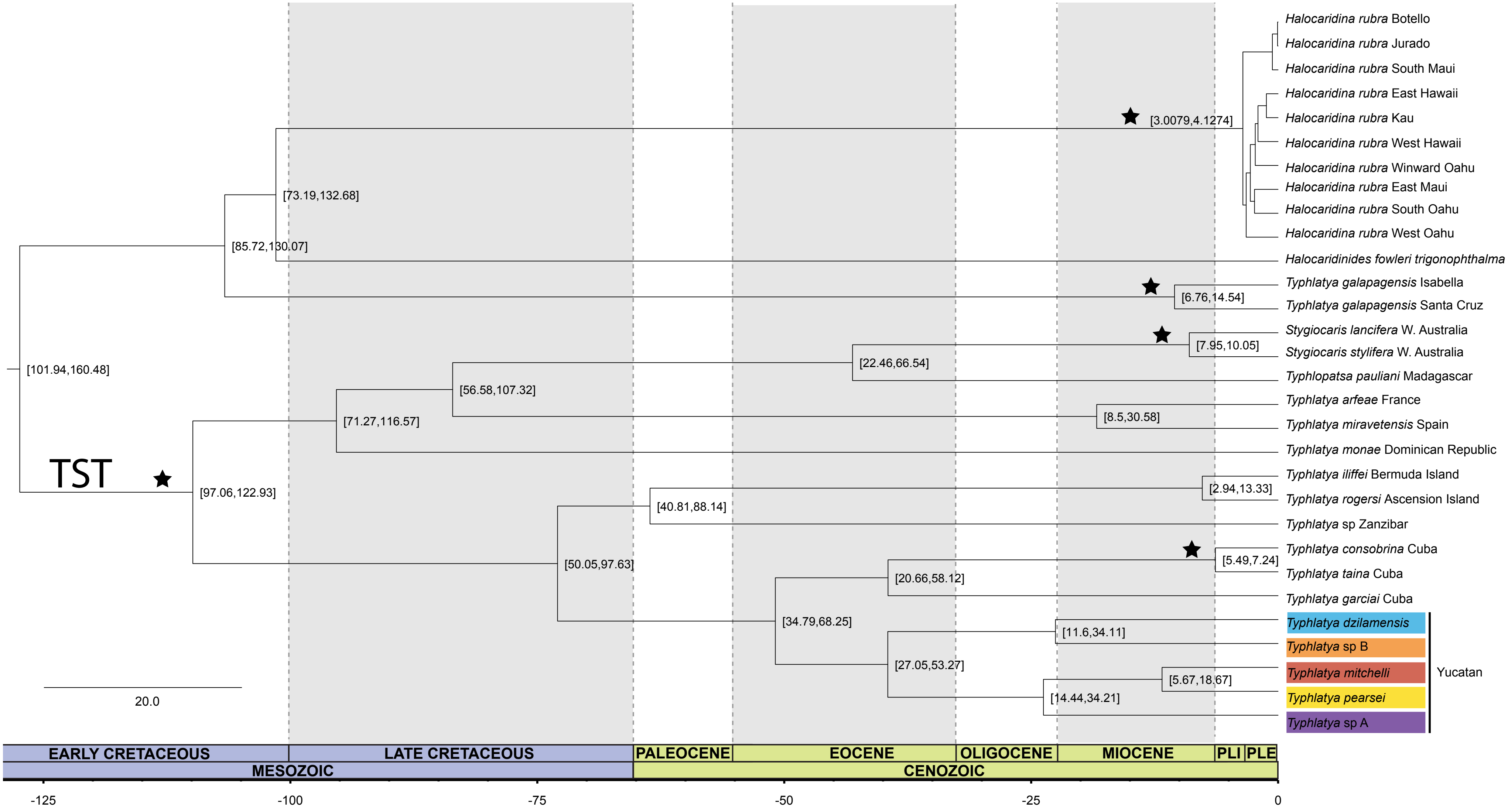

Halocaridina rubra Botello
Halocaridina rubra Jurado
Halocaridina rubra South Maui
Halocaridina rubra East Hawaii
Halocaridina rubra Kau
Halocaridina rubra West Hawaii
Halocaridina rubra Winward Oahu
Halocaridina rubra East Maui
Halocaridina rubra South Oahu
Halocaridina rubra West Oahu
Halocaridinides fowleri trigonophthalma
Typhlatya galapagensis Isabella
Typhlatya galapagensis Santa Cruz
Stygiocaris lancifera W. Australia
Stygiocaris stylifera W. Australia
Typhlopatsa pauliani Madagascar
Typhlatya arfeae France
Typhlatya miravetensis Spain
Typhlatya monae Dominican Republic
Typhlatya iliffei Bermuda Island
Typhlatya rogersi Ascension Island
Typhlatya sp Zanzibar
Typhlatya consobrina Cuba
Typhlatya taina Cuba
Typhlatya garciai Cuba
Typhlatya dzilamensis
Typhlatya sp B
Typhlatya mitchelli
Typhlatya pearsei
Typhlatya sp A
Yucatan
[3.0079,4.1274]
[73.19,132.68]
[85.72,130.07]
[6.76,14.54]
[7.95,10.05]
[22.46,66.54]
[56.58,107.32]
[71.27,116.57]
[8.5,30.58]
[101.94,160.48]
TST
[97.06,122.93]
[2.94,13.33]
[40.81,88.14]
[5.49,7.24]
[50.05,97.63]
[20.66,58.12]
[34.79,68.25]
[11.6,34.11]
[27.05,53.27]
[5.67,18.67]
[14.44,34.21]
20.0
EARLY CRETACEOUS
LATE CRETACEOUS
PALEOCENE
EOCENE
OLIGOCENE
MIOCENE
PLI
PLE
MESOZOIC
CENOZOIC
-125
-100
-75
-50
-25
0

v. SFigure 6.

SFigure 6. Chronogram showing divergence estimates [in brackets; Mya, 95% high posterior density limits (HPD) as confidence intervals], based on a relaxed, lognormal clock, Yule model and 4 gene partitions (COI+CYB, 1st codon / COI+CYB, 2nd codon / 16S / 28S +18S+H3, 1st+ 2nd codon) and 4 independent clock models for the *Late Cenozoic* clade. Stochastic mapping of salinity trait evolution is visualized with the probability density of each state at internal nodes. Circles at tips denote salinity preferences of lineages, low salinity (black; state 1) and high salinity (red; state 2). Uncertainty of ancestral states indicated at nodes.

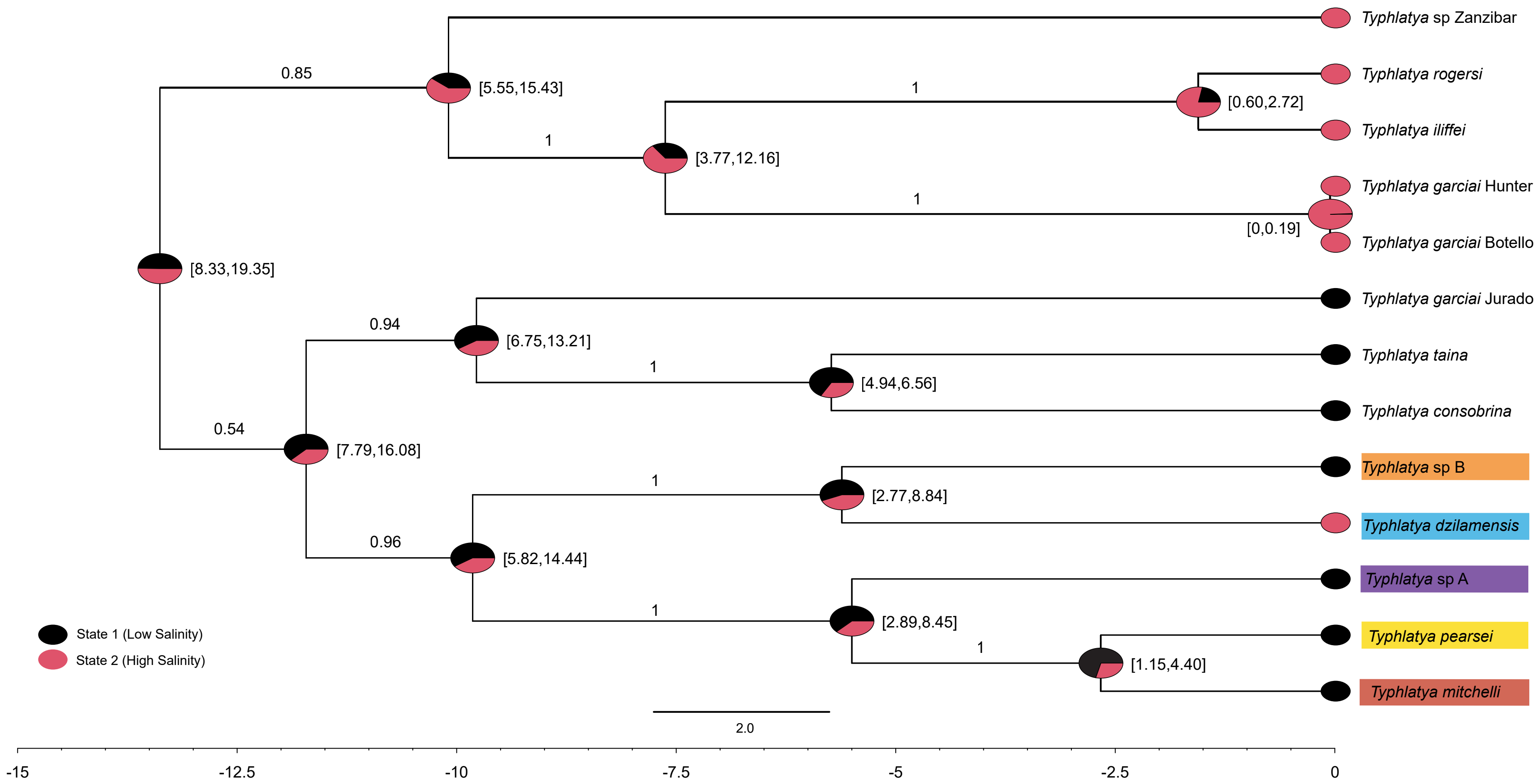
Typhlatya sp Zanzibar
Typhlatya rogersi
Typhlatya iliffei
Typhlatya garciai Hunter
Typhlatya garciai Botello
Typhlatya garciai Jurado
Typhlatya taina
Typhlatya consobrina
Typhlatya sp B
Typhlatya dzilamensis
Typhlatya sp A
Typhlatya pearsei
Typhlatya mitchelli
0.85
[5.55,15.43]
1
[0.60,2.72]
1
[3.77,12.16]
1
[0,0.19]
[8.33,19.35]
0.94
[6.75,13.21]
1
[4.94,6.56]
0.54
[7.79,16.08]
1
[2.77,8.84]
0.96
[5.82,14.44]
1
[2.89,8.45]
1
[1.15,4.40]
State 1 (Low Salinity)
State 2 (High Salinity)
2.0
-15
-12.5
-10
-7.5
-5
-2.5
0

vi. STable 1.

STable 1. Species delimitation analyses via GMYC, PTP, and bPTP methods. Both PTP and bPTP analyses exclude the outgroup.

Gene	GMYC: ML entities (Confidence Interval)	GMYC: Likelihood Ratio	GMYC: P	PTP Species Estimate	bPTP Species Estimate	bPTP Mean
**16S**	Yule: 6 (6-9)	Yule: 11.6802	Yule: 0.0029*	5	4-31	13.15
	Coalescent: 6 (5-10)	Coalescent: 9.5667	Coalescent: 0.0084*			
**CYTB**	Yule: 6 (6-7)	Yule: 14.4052	Yule: 0.0007*	5	5-12	5.4
	Coalescent: 6 (6-8)	Coalescent: 13.2861	Coalescent: 0.0013*			
**COI**	Yule: 6 (3-16)	Yule: 8.0347	Yule: 0.0180*	9	5-19	10.88
	Coalescent: 6 (3-16)	Coalescent: 7.8418	Coalescent: 0.0198*			
**6S+COI+CYT**	Yule: 6 (6-8)	Yule: 13.2418	Yule: 0.0013*	9	6-26	11.57
	Coalescent: 6 (6-8)	Coalescent: 22.40168	Coalescent: 1.3663e-05*			

vii. STable 2.

STable 2. Uncorrected pairwise (p) distances among Yucatán *Typhlatya* species. Interclade p-distances: 16S, below the diagonal and COI, above the diagonal. Intraclade p-distance along the diagonal, values listed as 16S/COI. -- indicates no value due single sequence availability.

	T. pearsei	***T. mitchelli***	***Typhlatya sp. A***	***T. dzilamensis***	***Typhlatya sp. B***	***Typhlatya c.f campecheae* "Cantemo"**
T. pearsei	*0.0066/0.0195*	0.1017	0.1520	0.1751	0.1795	0.0534
T. mitchelli	0.0539	*0.0058/0.0068*	0.1466	0.1691	0.1801	0.13
Typhlatya sp. A	0.1254	0.1279	*0.0117/0.0382*	0.1797	0.1950	0.1654
T. dzilamensis	0.1357	0.1455	0.1805	*0.0077/0.0074*	0.1417	0.1888
Typhlatya sp. B	0.1139	0.1317	0.1755	0.1110	*0.0060/--*	0.1897

viii. STable 3.

STable 3. Salinity and locality information for *Typhlatya* representatives included in this study. DNA ID follows STable 4. Locality indicates nearest city/town and state of the Yucatan Peninsula. [Note: Missing vouchers due to subsampling for stable isotope analyses in other studies[11,46]]

Taxon	DNA ID	Salinity (psu)	*Cenote*	Locality	Voucher
Typhlatya dzilamensis	14Bang01	2.16	Bang	Tulum, Quintana Roo	--
Typhlatya dzilamensis	17Tabanos02	3.96	Na’ach Wennen Ha (Tabanos)	Tulum, Quintana Roo	--
Typhlatya dzilamensis	19Tabanos04	3.96	Na’ach Wennen Ha (Tabanos)	Tulum, Quintana Roo	--
Typhlatya dzilamensis	18Tabanos03	3.96	Na’ach Wennen Ha (Tabanos)	Tulum, Quintana Roo	--
Typhlatya dzilamensis	6Crustacea11	13	Crustacea	Puerto Morelos, Quintana Roo	--
Typhlatya dzilamensis	9Crustacea14	13	Crustacea	Puerto Morelos, Quintana Roo	--
Typhlatya dzilamensis	10Crustacea16	13	Crustacea	Puerto Morelos, Quintana Roo	--
Typhlatya dzilamensis	23Crustacea50	13	Crustacea	Puerto Morelos, Quintana Roo	--
Typhlatya dzilamensis	20FDzilam01	33	Dzilam de Bravo	Dzilam, Yucatán	--
Typhlatya dzilamensis	15Odyssey02	33	Odyssey	Tulum, Quintana Roo	--
Typhlatya dzilamensis	7bCrustacea12	34	Crustacea	Puerto Morelos, Quintana Roo	--
Typhlatya dzilamensis	8Crustacea13	34	Crustacea	Puerto Morelos, Quintana Roo	--
Typhlatya dzilamensis	11Crustacea47	34	Crustacea	Puerto Morelos, Quintana Roo	--
Typhlatya dzilamensis	21Crustacea48	34	Crustacea	Puerto Morelos, Quintana Roo	--
Typhlatya dzilamensis	22Crustacea49	34	Crustacea	Puerto Morelos, Quintana Roo	--
Typhlatya dzilamensis	24Crustacea51	34	Crustacea	Puerto Morelos, Quintana Roo	--
Typhlatya dzilamensis	25Crustacea52	34	Crustacea	Puerto Morelos, Quintana Roo	--
Typhlatya dzilamensis	E4600	34	Sabtun 1	Chunchumil, Yucatan	--
Typhlatya dzilamensis	TX6	35	X'tabay	Akumal, Quintana Roo	--
Typhlatya dzilamensis	E4553	37	Cervera, Dzilam de Bravo	Dzilam, Yucatán	--
Typhlatya dzilamensis	27-1	--	27 Steps	Akumal, Quintana Roo	--
Typhlatya dzilamensis	AA3	--	Aayin Aak	Tulum, Quintana Roo	--
Typhlatya dzilamensis	TI013A	--	Actun Ha (Carwash)	Tulum, Quintana Roo	YUC-CC-255-11-006992
Typhlatya dzilamensis	28FBang07	--	Bang	Tulum, Quintana Roo	--
Typhlatya dzilamensis	Botello et al.	--	Cervera, Dzilam de Bravo	Dzilam, Yucatán	--
Typhlatya dzilamensis	ZMB DNA-604	--	Crustacea	Puerto Morelos, Quintana Roo	--
Typhlatya dzilamensis	Zaksek et al	--	Crustacea	Puerto Morelos, Quintana Roo	--
Typhlatya dzilamensis	Jurado-Rivera et al.	--	Dzilam de Bravo	Dzilam, Yucatán	--
Typhlatya dzilamensis	34Dzilam02	--	Dzilam de Bravo	Dzilam, Yucatán	--
Typhlatya dzilamensis	SM6	--	Santa Maria	Homun, Yucatán	--
Typhlatya dzilamensis	39b_Bang	--	Bang	Tulum, Quintana Roo	UNAM-CNCR-27969
Typhlatya dzilamensis	2_41_OdA	--	Odyssey	Tulum, Quintana Roo	UNAM-CNCR-27921
Typhlatya dzilamensis	2_50_OdA	--	Odyssey	Tulum, Quintana Roo	UNAM-CNCR-27925
Typhlatya dzilamensis	4_6_Tab	--	Na’ach Wennen Ha (Tabanos)	Tulum, Quintana Roo	UNAM-CNCR-28431
Typhlatya dzilamensis	Rr2_OdU	--	Odyssey	Tulum, Quintana Roo	
Typhlatya dzilamensis	4_35_TabA	--	Na’ach Wennen Ha (Tabanos)	Tulum, Quintana Roo	UNAM-CNCR-28390
Typhlatya dzilamensis	4_27_TabU	--	Na’ach Wennen Ha (Tabanos)	Tulum, Quintana Roo	UNAM-CNCR-28392
Typhlatya dzilamensis	4_3d_TabA	--	Na’ach Wennen Ha (Tabanos)	Tulum, Quintana Roo	UNAM-CNCR-28415
Typhlatya dzilamensis	3_29_OdU	--	Odyssey	Tulum, Quintana Roo	UNAM-CNCR-28581
Typhlatya dzilamensis	4_12_TabA	--	Na’ach Wennen Ha (Tabanos)	Tulum, Quintana Roo	UNAM-CNCR-28562
Typhlatya dzilamensis	Cervera	>30	Cervera, Dzilam de Bravo	Dzilam, Yucatán	--

Taxon	DNA ID	Salinity (psu)	*Cenote*	Locality	Voucher
Typhlatya mitchelli	TM42	0.63	Tza Itza	Tecoh, Yucatán	--
Typhlatya mitchelli	TM44	0.63	Tza Itza	Tecoh, Yucatán	--
Typhlatya mitchelli	13DRJailhouse03	2.9	Jailhouse	Tulum, Quintana Roo	--
Typhlatya mitchelli	CW2	--	Actun Ha (Carwash)	Tulum, Quintana Roo	--
Typhlatya mitchelli	CW3	--	Actun Ha (Carwash)	Tulum, Quintana Roo	--
Typhlatya mitchelli	CW4	--	Actun Ha (Carwash)	Tulum, Quintana Roo	--
Typhlatya mitchelli	CW6	--	Actun Ha (Carwash)	Tulum, Quintana Roo	--
Typhlatya mitchelli	von Rintelen et al.	--	Chac Mool	Playa del Carmen, Quintana Roo	--
Typhlatya mitchelli	Zaksek et al	--	Chihuán	Holca, Yucatán	--
Typhlatya mitchelli	CJ2	--	Chihuán	Holca, Yucatán	--
Typhlatya mitchelli	CJ3	--	Chihuán	Holca, Yucatán	--
Typhlatya mitchelli	CJ5	--	Chihuán	Holca, Yucatán	--
Typhlatya mitchelli	CJ6	--	Chihuán	Holca, Yucatán	--
Typhlatya mitchelli	CJ7	--	Chihuán	Holca, Yucatán	--
Typhlatya mitchelli	CJ8	--	Chihuán	Holca, Yucatán	--
Typhlatya mitchelli	E4671	--	Flor de Liz	Tixkokob, Yucatán	--
Typhlatya mitchelli	Botello et al.	--	Hoctun	Hoctun, Yucatan	--
Typhlatya mitchelli	K2	--	Kakuel	Mucuyche, Yucatán	--
Typhlatya mitchelli	K3	--	Kakuel	Mucuyche, Yucatán	--
Typhlatya mitchelli	K4	--	Kakuel	Mucuyche, Yucatán	--
Typhlatya mitchelli	K5	--	Kakuel	Mucuyche, Yucatán	--
Typhlatya mitchelli	K6	--	Kakuel	Mucuyche, Yucatán	--
Typhlatya mitchelli	CNCR 22696	--	--	--	--
Typhlatya mitchelli	N2	--	Naharon	La Veleta, Quintana Roo	--
Typhlatya mitchelli	SAC2	--	San Antonio	Cuzama, Yucatán	--
Typhlatya mitchelli	SAC3	--	San Antonio	Cuzama, Yucatán	--
Typhlatya mitchelli	SJ1	--	San Juan	Homun, Yucatán	--
Typhlatya mitchelli	SJ2	--	San Juan	Homun, Yucatán	--
Typhlatya mitchelli	SJ3	--	San Juan	Homun, Yucatán	--
Typhlatya mitchelli	SJ4	--	San Juan	Homun, Yucatán	--
Typhlatya mitchelli	SJ11	--	San Juan	Homun, Yucatán	--
Typhlatya mitchelli	SJ12	--	San Juan	Homun, Yucatán	--
Typhlatya mitchelli	SJ14	--	San Juan	Homun, Yucatán	--
Typhlatya mitchelli	von Rintelen et al.	--	San Juan	Homun, Yucatán	--
Typhlatya mitchelli	TI008D	--	Sistema Paamul	Paamul, Quintana Roo	YUC-CC-255-11-006995
Typhlatya mitchelli	3_51_OdA	--	Odyssey	Tulum, Quintana Roo	UNAM-CNCR-28589
Typhlatya pearsei	TP12	0.67	Noh-Mozon	Pixya, Yucatán	--
Typhlatya pearsei	12Jailhouse01	3.5	Jailhouse	Tulum, Quintana Roo	--
Typhlatya pearsei	C1	--	Grutas de Xtacumbilxunaan	Bolonchén, Campeche	YUC-CC-255-11-006998
Typhlatya pearsei	C2	--	Grutas de Xtacumbilxunaan	Bolonchén, Campeche	YUC-CC-255-11-006999
Typhlatya pearsei	C3	--	Grutas de Xtacumbilxunaan	Bolonchén, Campeche	YUC-CC-255-11-007015
Typhlatya pearsei	MLP85.1	--	--	--	--
Typhlatya pearsei	Botello et al.	--	Nohchen	Sacalum, Yucatán	--
Typhlatya pearsei	Jurado-Rivera et al.	--	Nohchen	Sacalum, Yucatán	--
Typhlatya pearsei	TI166A	--	Sabak Ha	Sacalum, Yucatán	YUC-CC-255-11-007000
Typhlatya pearsei	SAY2	--	San Antonio Chiich	Yokdzonot, Yucatán	--
Typhlatya pearsei	SAY3	--	San Antonio Chiich	Yokdzonot, Yucatán	--
Typhlatya pearsei	SAY4	--	San Antonio Chiich	Yokdzonot, Yucatán	--
Typhlatya pearsei	SAY5	--	San Antonio Chiich	Yokdzonot, Yucatán	--
Typhlatya pearsei	SAY6	--	San Antonio Chiich	Yokdzonot, Yucatán	--
Typhlatya pearsei	TI008B	--	Sistema Paamul	Paamul, Quintana Roo	--
Typhlatya pearsei	3_47_OdA	--	Odyssey	Tulum, Quintana Roo	UNAM-CNCR-28590
Typhlatya pearsei	3_12_OdA	--	Odyssey	Tulum, Quintana Roo	UNAM-CNCR-28585
Typhlatya pearsei	Yucatán	--	--	Yucatán	--
Typhlatya pearsei	Cantemo	--	Cantemo	Cantemo, Campeche	--

**Taxon**	**DNA ID**	**Salinity (psu)**	***Cenote***	**Locality**	Voucher
Typhlatya sp. A	33FBang14	2.5	Bang	Tulum, Quintana Roo	--
Typhlatya sp. A	32FBang13	2.5	Bang	Tulum, Quintana Roo	--
Typhlatya sp. A	TI172	--	Kankirixche	Abalá, Yucatán	YUC-CC-255-11-007002
Typhlatya sp. A	TI157B	--	Nah-Yah	Pixya, Yucatán	--
Typhlatya sp. A	Rc1_OdU	--	Odyssey	Tulum, Quintana Roo	--
Typhlatya sp. A	3_11_OdU	--	Odyssey	Tulum, Quintana Roo	UNAM-CNCR-28588
Typhlatya sp. A	3_32_OdU	--	Odyssey	Tulum, Quintana Roo	UNAM-CNCR-28565
Typhlatya sp. B	16Tabanos01	4.38	Na'ach Wennen Ha (Tabanos)	Tulum, Quintana Roo	--
Typhlatya sp. B	27FBang06	--	Bang	Tulum, Quintana Roo	--
Typhlatya sp. B	ZMB DNA-600	--	Hoctun	Hoctun, Yucatan	--
Typhlatya sp. B	4_9_TabA	--	Na'ach Wennen Ha (Tabanos)	Tulum, Quintana Roo	UNAM-CNCR-28563
Typhlatya sp. B	4_32_TabA	--	Na'ach Wennen Ha (Tabanos)	Tulum, Quintana Roo	UNAM-CNCR-28586
Typhlatya sp. B	4_41_TabA	--	Na'ach Wennen Ha (Tabanos)	Tulum, Quintana Roo	UNAM-CNCR-28564

ix. STable 4.

STable 4. Taxonomic sampling, GenBank species identities from previous studies, DNA identifier, GenBank accession numbers, and GenBank References for *Typhlatya* representatives included in this study. Taxon names revised according to current phylogenic hypotheses (Figure 4).

Taxon	GenBank ID*	DNA ID	CO1	CYTB	16S	H3	18S	28S	Reference
Typhlatya dzilamensis	*Typhlatya pearsei*	27-1	AY115535	AY115532	AY115538				Hunter et al., 2008
Typhlatya dzilamensis	*Typhlatya pearsei*	AA3	AY115534	AY115531	AY115537				Hunter et al., 2008
Typhlatya dzilamensis		TI013A	OM456520	OM455439	OM458915	OM455426	OM458838	OM458884	This Study
Typhlatya dzilamensis		28FBang07			OM458943				This Study
Typhlatya dzilamensis		14Bang01			OM458930				This Study
Typhlatya dzilamensis	*Typhlatya dzilamensis*	Botello et al.	HE800926	HE800951	HE800997	HE800972			Botello et al., 2012
Typhlatya dzilamensis		E4553		OM455435	OM458910	OM455421	OM458832		This Study
Typhlatya dzilamensis	*Typhlatya* sp.	ZMB DNA-604			FN995396	FN995541		FN995630	von Rintelen et al., 2012
Typhlatya dzilamensis	*Typhlatya pearsei*	Zaksek et al.						DQ641628	Zaksek et al., 2007
Typhlatya dzilamensis		6Crustacea11			OM458922				This Study
Typhlatya dzilamensis		7bCrustacea12			OM458923				This Study
Typhlatya dzilamensis		8Crustacea13			OM458924				This Study
Typhlatya dzilamensis		9Crustacea14			OM458925				This Study
Typhlatya dzilamensis		10Crustacea16			OM458926				This Study
Typhlatya dzilamensis		11Crustacea47			OM458927				This Study
Typhlatya dzilamensis		21Crustacea48			OM458937				This Study
Typhlatya dzilamensis		22Crustacea49			OM458938				This Study
Typhlatya dzilamensis		23Crustacea50			OM458939				This Study
Typhlatya dzilamensis		24Crustacea51			OM458940				This Study
Typhlatya dzilamensis		25Crustacea52			OM458941				This Study
Typhlatya dzilamensis	*Typhlatya dzilamensis*	Jurado-Rivera et al.	KX844719	KX844719	KX844719				Jurado-Rivera et al., 2017
Typhlatya dzilamensis		34Dzilam02			OM458946				This Study
Typhlatya dzilamensis		20FDzilam01			OM458936				This Study
Typhlatya dzilamensis		17Tabanos02			OM458933				This Study
Typhlatya dzilamensis		19Tabanos04			OM458935				This Study
Typhlatya dzilamensis		18Tabanos03			OM458934				This Study
Typhlatya dzilamensis		15Odyssey02			OM458931				This Study
Typhlatya dzilamensis		E4600			OM458911	OM455422	OM458833	OM458880	This Study
Typhlatya dzilamensis	*Typhlatya pearsei*	SM6	AY115536	AY115533	AY115539				Hunter et al., 2008
Typhlatya dzilamensis		TX6		OM455443	OM458921	OM455432			This Study
Typhlatya dzilamensis	*Typhlatya dzilamensis*	39b_Bang	OM456529						This Study, Chavez-Diaz et al, 2018
Typhlatya dzilamensis	*Typhlatya dzilamensis*	2_41_OdA	OM456526						This Study, Chavez-Diaz et al, 2018
Typhlatya dzilamensis	*Typhlatya dzilamensis*	2_50_OdA	OM456527						This Study, Chavez-Diaz et al, 2018
Typhlatya dzilamensis	*Typhlatya dzilamensis*	4_6_Tab	OM456534						This Study, Chavez-Diaz et al, 2018
Typhlatya dzilamensis	*Typhlatya dzilamensis*	Rr2_OdU	OM456536						This Study, Chavez-Diaz et al, 2018
Typhlatya dzilamensis	*Typhlatya dzilamensis*	4_35_TabA	OM456532						This Study, Chavez-Diaz et al, 2018
Typhlatya dzilamensis	*Typhlatya dzilamensis*	4_27_TabU	OM456531						This Study, Chavez-Diaz et al, 2018
Typhlatya dzilamensis	*Typhlatya dzilamensis*	4_3d_TabA	OM456533						This Study, Chavez-Diaz et al, 2018
Typhlatya dzilamensis	*Typhlatya dzilamensis*	3_29_OdU	OM456528						This Study, Chavez-Diaz et al, 2018
Typhlatya dzilamensis	*Typhlatya dzilamensis*	4_12_TabA	OM456530						This Study, Chavez-Diaz et al, 2018
Typhlatya dzilamensis	*Typhlatya dzilamensis*	Cervera	OM456535						This Study, Chavez-Diaz et al, 2019

Taxon	GenBank ID*	DNA ID	CO1	CYTB	16S	H3	18S	28S	Reference
Typhlatya mitchelli	*Typhlatya mitchelli*	CW2	AF513510	AF512033	AF513525				Hunter et al., 2008
Typhlatya mitchelli	*Typhlatya mitchelli*	CW3	AF513511	AF512034	AF513526				Hunter et al., 2008
Typhlatya mitchelli	*Typhlatya mitchelli*	CW4	AF513512	AF512035	AF513527				Hunter et al., 2008
Typhlatya mitchelli	*Typhlatya mitchelli*	CW6		AF512036					Hunter et al., 2008
Typhlatya mitchelli	*Typhlatya* sp.	ZMB DNA-603			FN995395	FN995540		FN995629	von Rintelen et al., 2012
Typhlatya mitchelli	*Typhlatya mitchelli*	Zaksek et al.						DQ641629	Zaksek et al., 2007
Typhlatya mitchelli	*Typhlatya mitchelli*	CJ2	AF513513	AF512037	AF513528				Hunter et al., 2008
Typhlatya mitchelli	*Typhlatya mitchelli*	CJ3	AF513514	AF512038	AF513529				Hunter et al., 2008
Typhlatya mitchelli	*Typhlatya mitchelli*	CJ5	AF513515	AF512039	AF513530				Hunter et al., 2008
Typhlatya mitchelli	*Typhlatya mitchelli*	CJ6		AF512040					Hunter et al., 2008
Typhlatya mitchelli	*Typhlatya mitchelli*	CJ7		AF512041					Hunter et al., 2008
Typhlatya mitchelli	*Typhlatya mitchelli*	CJ8		AF512042					Hunter et al., 2008
Typhlatya mitchelli	*Typhlatya mitchelli*	E4671		OM455436	OM458912	OM455423	OM458834	OM458881	This Study
Typhlatya mitchelli	*Typhlatya mitchelli*	Botello et al.	HE800902/HE800924	HE800949	HE800995	HE800970	HE801021	HE801041	Botello et al., 2012
Typhlatya mitchelli	*Typhlatya mitchelli*	13DRJailhouse03	--	--	OM458929	--	--	--	This Study
Typhlatya mitchelli	*Typhlatya mitchelli*	K2	AF513521	AF512052	AF513536				Hunter et al., 2008
Typhlatya mitchelli	*Typhlatya mitchelli*	K3	AF513522	AF512053	AF513537				Hunter et al., 2008
Typhlatya mitchelli	*Typhlatya mitchelli*	K4	AF513523	AF512054	AF513538				Hunter et al., 2008
Typhlatya mitchelli	*Typhlatya mitchelli*	K5		AF512055					Hunter et al., 2008
Typhlatya mitchelli	*Typhlatya mitchelli*	K6		AF512056					Hunter et al., 2008
Typhlatya mitchelli	*Typhlatya mitchelli*	CNCR 22696			EU868644		EU868735		Bracken et al., 2009
Typhlatya mitchelli	*Typhlatya mitchelli*	N2	AF513509	AF512032	AF513524				Hunter et al., 2008
Typhlatya mitchelli	*Typhlatya mitchelli*	SAC2	AF513516	AF512043	AF513531				Hunter et al., 2008
Typhlatya mitchelli	*Typhlatya mitchelli*	SAC3	AF513517	AF512044	AF513532				Hunter et al., 2008
Typhlatya mitchelli	*Typhlatya mitchelli*	SJ1	AF513518	AF512045	AF513533				Hunter et al., 2008
Typhlatya mitchelli	*Typhlatya mitchelli*	SJ2	AF513519	AF512046	AF513534				Hunter et al., 2008
Typhlatya mitchelli	*Typhlatya mitchelli*	SJ3	AF513520	AF512047	AF513535				Hunter et al., 2008
Typhlatya mitchelli	*Typhlatya mitchelli*	SJ4		AF512048					Hunter et al., 2008
Typhlatya mitchelli	*Typhlatya mitchelli*	SJ11		AF512049					Hunter et al., 2008
Typhlatya mitchelli	*Typhlatya mitchelli*	SJ12		AF512050					Hunter et al., 2008
Typhlatya mitchelli	*Typhlatya mitchelli*	SJ14		AF512051					Hunter et al., 2008
Typhlatya mitchelli	*Typhlatya mitchelli*	von Rintelen et al.			FN995393	FN995538		FN995627	von Rintelen et al., 2012
Typhlatya mitchelli	*Typhlatya mitchelli*	TI008D	OM456519	OM455438	OM458914	OM455425	OM458837	OM458883	This Study
Typhlatya mitchelli	*Typhlatya mitchelli*	TM42	OM456523		OM458919	OM455430	OM458841	OM458888	This Study
Typhlatya mitchelli	*Typhlatya mitchelli*	TM44	OM456524	OM455441		OM455431	OM458842	OM458889	This Study
Typhlatya mitchelli	*Typhlatya mitchelli*	3_51_OdA	OM456537						This Study, Chavez-Diaz et al, 2018

Taxon	GenBank ID*	DNA ID	CO1	CYTB	16S	H3	18S	28S	Reference
Typhlatya pearsei	*Typhlatya campecheae*	C1		OM455433	OM458907		OM458835		This Study
Typhlatya pearsei	*Typhlatya campecheae*	C2		OM455434	OM458908	OM455419	OM458836	OM458878	This Study
Typhlatya pearsei	*Typhlatya campecheae*	C3			OM458909	OM455420		OM458879	This Study
Typhlatya pearsei		12Jailhouse01			OM458928				This Study
Typhlatya pearsei	*Typhlatya pearsei*	MLP85.1			DQ079735	DQ079702	DQ079770	DQ079813	Porter et al., 2005
Typhlatya pearsei		TP12	OM456525	OM455442	OM458920			OM458890	This Study
Typhlatya pearsei	*Typhlatya pearsei*	Botello et al.	HE800903/HE800925	HE800950	HE800996	HE800971	HE801022	HE801042	Botello et al., 2012
Typhlatya pearsei	*Typhlatya pearsei*	Jurado-Rivera et al.	KX844709	KX844709	KX844709				Jurado-Rivera et al., 2017
Typhlatya pearsei		TI166A	OM456522		OM458917	OM455428		OM458886	This Study
Typhlatya pearsei	*Typhlatya* sp.	SAY2		AY115545	AY115540				Hunter et al., 2008
Typhlatya pearsei	*Typhlatya* sp.	SAY3		AY115546	AY115541				Hunter et al., 2008
Typhlatya pearsei	*Typhlatya* sp.	SAY4		AY115547	AY115542				Hunter et al., 2008
Typhlatya pearsei	*Typhlatya* sp.	SAY5		AY115548	AY115543				Hunter et al., 2008
Typhlatya pearsei	*Typhlatya* sp.	SAY6		AY115549	AY115544				Hunter et al., 2008
Typhlatya pearsei		TI008B	OM456518	OM455437	OM458913	OM455424		OM458882	This Study
Typhlatya pearsei		3_47_OdA	OM456539						This Study, Chavez-Diaz et al, 2018
Typhlatya pearsei		3_12_OdA	OM456538						This Study, Chavez-Diaz et al, 2018
Typhlatya pearsei	*Typhlatya pearsei*	Yucatan	OM456540						This Study, Chavez-Diaz et al, 2018
Typhlatya pearsei	*Typhlatya campecheae*	Cantemo	OM456541						This Study, Chavez-Diaz et al, 2018
Typhlatya sp. A		33FBang14			OM458945				This Study
Typhlatya sp. A		32FBang13			OM458944				This Study
Typhlatya sp. A		TI172		OM455440	OM458918	OM455429	OM458840	OM458887	This Study
Typhlatya sp. A		TI157B	OM456521		OM458916	OM455427	OM458839	OM458885	This Study
Typhlatya sp. A	*Typhlatya* sp. 2	Rc1_OdU	OM456544						This Study, Chavez-Diaz et al, 2018
Typhlatya sp. A	*Typhlatya* sp. 2	3_11_OdU	OM456542						This Study, Chavez-Diaz et al, 2018
Typhlatya sp. A	*Typhlatya* sp. 2	3_32_OdU	OM456543						This Study, Chavez-Diaz et al, 2018
Typhlatya sp. B		27FBang06			OM458942				This Study
Typhlatya sp. B	*Typhlatya mitchelli*	ZMB DNA-600	KX844712	KX844712	KX844712				Jurado-Rivera et al., 2017
Typhlatya sp. B		16Tabanos01			OM458932				This Study
Typhlatya sp. B	*Typhlatya* sp. 1	4_9_TabA	OM456547						This Study, Chavez-Diaz et al, 2018
Typhlatya sp. B	*Typhlatya* sp. 1	4_32_TabA	OM456545						This Study, Chavez-Diaz et al, 2018
Typhlatya sp. B	*Typhlatya* sp. 1	4_41_TabA	OM456546						This Study, Chavez-Diaz et al, 2018
Typhlatya consobrina		Jurado-Rivera et al.	KX844717	KX844717	KX844717				Jurado-Rivera et al., 2017
Typhlatya consobrina		Botello et al.				HE800979	HE801028	HE801048	Botello et al., 2012
Typhlatya garciai		Jurado-Rivera et al.	KX844720	KX844720	KX844720				Jurado-Rivera et al., 2017
Typhlataya taina		Botello et al.				HE800980	HE801029	HE801049	Botello et al., 2012
Typhlatya taina		Jurado-Rivera et al.	KX844708	KX844708	KX844708				Jurado-Rivera et al., 2017

x. STable 5

STable 5. (A) Divergence date estimates and parameters for Yucatán and Cuba Typhlatya sister clades based on the Yule model. Median heights (Myr), 95% high posterior densities (HPD), Mean likelihood, Mean Effective Sample Size (ESS), and mean Ages (mya) of the Most Recent Common Ancestor (mcra) for calibration points (following Botello et al., 2013; and/or Jurado et al. 2017. See Text and STable 7 for details); (B) Comparison of mcraAge(Mry) estimates of Yucatan and Cuba clade members from previous work (Jurado-Rivera et al., 2017, Botello et al., 2013) and the current study.

**A.**	**Late Cenozoic Clade**						**Anchialine Atyidae Clade**					
	Relaxed			Strict			Relaxed					
	4 Clocks			2 clocks			5 clocks					
	4 Partitions, no 3rd codon			4 Partitions, no 3rd codon			5 Partitions, no 3rd codon					
	**Height**	**95% HPD**		**Height**	**95% HPD**		**Height**	**95% HPD**		**Height**	**95% HPD**	
	Mean	Upper	Lower	Mean	Upper	Lower	Mean	Upper	Lower	Mean	Upper	Lower
Yucatán *Typhlatya*	9.82	12.43	5.82	11.25	18.69	10.89	14.7	18.69	10.89	39.52	53.27	27.05
Typhlatya sp. A + *(T. mitchelli + T. pearsei)*	5.5	8.45	2.89	5.72	12.85	6.544	9.56	12.85	6.544	23.76	34.21	12.44
T. mitchelli + T. pearsei	2.67	4.39	1.15	2.75	7.04	2.74	4.81	7.04	2.74	11.75	18.67	5.67
T. dzilamensis + Typhlatya sp. B	5.62	8.84	2.77	6.98	11.81	5.23	8.49	11.81	5.23	22.54	34.11	11.59
Cuba *Typhlatya*	9.78	13.21	6.75	10.62	19.76	11.03	15.25	19.76	11.03	39.49	58.12	20.66
T. taina + T. consobrina	5.74	6.56	4.94	5.79	7.01	5.34	6.15	7.01	5.34	6.35	7.24	5.49
Yucatán *Typhlatya* + Cuba *Typhlatya*	11.71	16.08	7.79	10.33	23.34	14.19	18.57	23.34	14.19	50.9	68.25	34.78
Mean Log Likelihood		-14864.3287			-15182.53			-30572.64			-30569.56	
ESS		54669.2			49692.7			24849.9			5055.6	
mcraAge (*Halocaridina*)		--			--			2.85			3.57	
mcraAge *(Stygiocaris)*		--			--			8.62			8.99	
mcraAge *(T. taina + T. consobrina)*		5.74			5.79			6.15			6.35	
mcraAge *(T. galapagensis)*		--			--			4.62			10.49	
Calibration Prior		*T. taina + T. consobrina*			*T. taina + T. consobrina*			Geological (4)			Geological + Fossil (5)	

**B.**	**Jurado-Rivera et al., 2017**			**Botello et al, 2013**			**Cenozoic Clade**			**Anchialine Atyidae Clade**		
	**Height**	**95% HPD**		**Height**	**95% HPD**		**Height**	**95% HPD**		**Height**	**95% HPD**	
**mcraAge**	Mean	Upper	Lower	Mean	Upper	Lower	Mean	Upper	Lower	Mean	Upper	Lower
Yucatán *Typhlatya*	56.50	70.07	43.11	14.91	20.22	9.70	9.82	12.43	5.82	14.7	18.69	10.89
Typhlatya sp. A + *T. mitchelli + T. pearsei*	--	--	--	--	--	--	5.5	8.45	2.89	9.56	12.85	6.544
T. mitchelli + T. pearsei	--	--	--	4.70	6.90	2.70	2.67	4.39	1.15	4.81	7.04	2.74
T. dzilamensis + Typhlatya. sp. B	33.35	44.89	22.36	--	--	--	5.62	8.84	2.77	8.49	11.81	5.23
T. garciai + (*T. taina + T. consobrina*)	--	--	--	--	--	--	9.78	13.21	6.75	15.25	19.76	11.03
T. taina + T. consobrina	43.52	58.66	29.25	5.52	5.99	5.05	5.74	6.56	4.94	6.15	7.01	5.34
Yucatán *Typhlatya* + Cuba *Typhlatya*	74.01	88.61	60.08	20.12	26.54	14.24	11.71	16.08	7.79	18.57	23.34	14.19

xi. STable 6

STable 6. Node age estimates based on alternate priors used for node calibration (See STable 7 for dates used as prior).

	TST + All			TST only			*Halocaridina* only		
	**Height**	**95% HPD**		**Height**	**95% HPD**		**Height**	**95% HPD**	
	Mean	Upper	Lower	Mean	Upper	Lower	Mean	Upper	Lower
Yucatán *Typhlatya*	39.52	53.27	27.05	67.36	81.88	53.59	22.66	30.97	15.12
Typhlatya sp. A + *(T. mitchelli + T. pearsei)*	23.76	34.21	12.44	43.37	56.57	31.03	14.59	20.68	8.96
T. mitchelli + T. pearsei	11.75	18.67	5.67	21.41	30.78	12.87	7.26	10.99	3.99
T. dzilamensis + Typhlatya sp. B	22.54	34.11	11.59	39.98	53.7	27.18	13.31	19.58	7.67
Cuba *Typhlatya*	39.49	58.12	20.66	70.76	88.16	54.43	23.88	32.82	15.73
T. taina + T. consobrina	6.35	7.24	5.49	33.33	48.24	23.48	11.94	17.28	7.02
Yucatán *Typhlatya* + Cuba *Typhlatya*	50.9	68.25	34.78	84.5	101.1	68.95	28.44	38.38	19.38
Mean Log Likelihood		-30569.56			-30571.7565			-30571.9661	
ESS		5055.6			15105.4			13044.4	
mcraAge (*Halocaridina*)		**3.57**			8.25			**3.13**	
mcraAge *(Stygiocaris)*		**8.99**			43.56			14.59	
mcraAge *(T. taina + T. consobrina)*		**6.35**			35.33			11.94	
mcraAge (T. galapagensis)		**10.49**			21.31			7.27	
mcraAge (TST)		**109.86**			**128.85**			42.61	

	Stygiocaris only			*T. galapagensis* only			*T. taina+T. consobrina* only		
	**Height**	**95% HPD**		**Height**	**95% HPD**		**Height**	**95% HPD**	
	Mean	Upper	Lower	Mean	Upper	Lower	Mean	Upper	Lower
Yucatán *Typhlatya*	11.56	15.53	7.92	13.53	22.25	6.18	9.51	12.89	6.46
Typhlatya sp. A + *(T. mitchelli + T. pearsei)*	7.43	10.47	4.71	8.73	14.84	3.84	6.11	8.66	3.81
T. mitchelli + T. pearsei	3.68	5.5	1.98	4.31	7.6	1.69	3.02	4.59	1.6
T. dzilamensis + Typhlatya sp. B	6.85	9.78	4.16	8.04	13.74	3.37	5.59	8.22	3.3
Cuba *Typhlatya*	12.15	16.5	8.11	14.2	23.32	6.28	10.24	13.54	7.36
T. taina + T. consobrina	6.07	8.77	3.55	7.12	12.07	2.91	5.7	6.52	4.92
Yucatán *Typhlatya* + Cuba *Typhlatya*	14.47	19.32	10.15	16.95	27.67	7.8	11.98	16.01	8.46
Mean Log Likelihood		-30571.67			-30571.54			30571.43	
ESS		13881.9			13361.8			13267.5	
mcraAge (*Halocaridina*)		1.42			1.65			1.15	
mcraAge *(Stygiocaris)*		**8.2785**			8.72			6.03	
mcraAge *(T. taina + T. consobrina)*		6.85			7.12			**5.7**	
mcraAge (T. galapagensis)		3.68			**5.5745**			3.02	
mcraAge (TST)		21.87			25.5			17.71	

xii. STable 7.

STable 7. Summary of model selection process for BEAST analyses under the Yule diversification model.

Clade	Number of Clocks	Clock Models	Marginal Likelihood	Bayes Factor	Number of Partitions
**Cenozoic (no3rd)**	**4**	**UCLN**	**-14975.37**	**0**	**4**
Cenozoic (no3rd)	2	Strict	-15061.13	171.52	4
Cenozoic (no3rd)	2	UCLN	-15268.14	585.54	4
Cenozoic (no3rd)p	2	UCLN	-15269.5	588.26	4
Cenozoic (no3rd)	4	Strict	-15436.41	922.08	4
Cenozoic (no3rd)	1	UCLN	-23862.04	17773.34	4
Cenozoic	1	UCLN	-23862.04	17773.34	6
Cenozoic	2	UCLN	-23862.51	17774.28	6
Cenozoic	6	UCLN	-23902.25	17853.76	6
Cenozoic	1	Strict	-23964	17977.26	6
Cenozoic	2	Strict	-23967.8	17984.86	6
Cenozoic (no3rd)	1	Strict	-23986.8	18022.86	4
Cenozoic	6	Strict	-24534.12	19117.5	6
**Atyidae (no3rd)**	**5**	**UCLN**	**-31088.58**	**0**	**5**
Atyidae (no3rd)	2	UCLN	-31297.8	418.44	5
Atyidae (no3rd)	5	Strict	-31587.46	997.76	5
Atyidae (no3rd)	2	Strict	-31590.09	1003.02	5
Atyidae	7	Strict	-31601.16	1025.16	7
Atyidae (no3rd)	1	UCLN	-31744.58	1312	5
Atyidae (no3rd)	1	Strict	-31918.19	1659.22	5
Atyidae	7	UCLN	-49742.72	37308.28	7
Atyidae	2	UCLN	-51042.49	39907.82	7
Atyidae	2	Strict	-51326.7	40476.24	7
Atyidae	1	UCLN	-52064.64	41952.12	7
Atyidae	1	Strict	-52348.85	55629.46	7

Partition Scheme (Cenozoic)

6 Partition	Model	4 Partition	Model
(1) COI+CYTB, 1st	TIM2e+G4	(1) COI+CYTB, 1st	TIM2e+G4
(2) COI+CYTB, 2nd	K3Pu+F+I	(2) COI+CYTB, 2nd	K3Pu+F+I
(3) COI+CYTB, 3rd	TIM+F+G4	(3) 16S	TN+F+I
(4) 16S	TN+F+I	(4) 28S+18S+H3 (1st+2nd)	GTR+F+I+G4
(5) 28S+18S+H3, 1st+2n	GTR+F+I+G4		
(6) H3, 3rd	GTR+F+I+G4		

Calibration Date Prior:
T. taina + T. consobrina, ~5-7 MYA

Partition Scheme (Atyidae)

7 Partition	Model	5 Partition	Model
(1) COI+CYTB+H3, 1st	TIM2e+I+G4	(1) COI+CYTB+H3, 1st	TIM2e+I+G4
(2) COI+CYTB, 2nd	TIM2e+I+G4	(2) COI+CYTB, 2nd	TIM2e+I+G4
(3) COI+CYTB, 3rd	GTR+F+I+G4	(3) 16S	TIM3+F+I+G4
(4) 16S	TIM3+F+I+G4	(4) 18S+H3, 2nd	K2P+I
(5) 18S+H3, 2nd	K2P+I	(5) 28S	TIM3+F+I+G4
(6) 28S	TIM3+F+I+G4		
(7) H3, 3rd	TVMe+G4		

Calibration Date Priors:
TST: ~118-149 MYA (Fossil)
Halocaridina: ~2-4 MYA (Geological) T. galapagensis, ~5-14 MYA (Geological)
Stygiocaris: ~7-10 MYA (Geological) T. taina + T. consobrina, ~5-7 MYA (Geological)

Clade refers to phylogenies presented in Figure 6 and SFigure 5 and 6. Gene partition scheme (6 and 7) and substitution model selection based on PartitionFinder via IQ-Tree. Gene partitions schemes 4 and 5 exclude 3rd codon position for protein coding genes (COI, CYTB, H3). Number of Clocks: 1, single clock model for all gene partitions (all linked); 2, two clock models linking mitochondrial (COI, CYTB, 16S) and nuclear (18S, 28S, H3) gene partitions, respectively; 4, 5, 6 and 7, four, five, six and seven clock models according to respecitive partitions. [Exception: Partitions 5 and 7 include H3, 1st codon with COI+CYTB, 1st codon partiion]. UCLN: Uncorrelated log-normal clock model. Strict: Strict clock model. Calibration data priors based on Botello et al., 2013 and Jurado-Rivera et al., 2017.

xiii. STable 8.

STable 8. Updated list of the Typhlatya diversity and distribution, following Botello et al., 2013.

*New to this study.

Species	Distribution	Region
T. kakuki Alvarez et al., 2005	Acklins (Bahamas)	Caribbean
Typhlatya sp.	Aruba (Netherlands Antilles)	Caribbean
T. rogersi Chace & Manning, 1972	Ascension Island	Atlantic
Typhlatya sp.	Belize	Caribbean
T. iliffei Hart & Manning, 1981	Bermuda	Atlantic
Typhlatya sp.	Bonaire (Netherlands Antilles)	Caribbean
T. consobrina Botosaneanu & Holthuis, 1970	Cuba	Caribbean
T. elenae Juarrero, 1994	Cuba	Caribbean
T. garciadebrasi Juarrero & Ortiz, 2000	Cuba	Caribbean
T. taina Estrada & Go´ mez, 1987	Cuba	Caribbean
T. garciai Chace, 1942	Cuba; Caicos Islands	Caribbean
T. arfeae Jaume & Bre´hier, 2005	France	Mediterranean
T. monae Chace, 1954	Puerto Rico; Dominican Republic; Mona Island (Puerto Rico); Barbuda (Lesser Antilles); Curacao (Netherlands Antilles); San Andre´s Island (Colombia)	Caribbean
T. galapagensis Monod & Cals, 1970	Santa Cruz and Isabela Islands (Galapagos)	Pacific
T. miravetensis Sanz & Platvoet, 1995	Spain	Mediterranean
T. utilaensis Alvarez et al., 2005	Utila Island (Honduras)	Caribbean
Typhlatya sp. A*	Yucatan Peninsula (Mexico)	Caribbean
Typhlatya sp. B*	Yucatan Peninsula (Mexico)	Caribbean
T. campechae Hobbs & Hobbs, 1976	Yucatan Peninsula (Mexico)	Caribbean
T. dzilamensis Alvarez et al., 2005	Yucatan Peninsula (Mexico)	Caribbean
T. mitchelli Hobbs & Hobbs, 1976	Yucatan Peninsula (Mexico)	Caribbean
T. pearsei Creaser, 1936	Yucatan Peninsula (Mexico)	Caribbean

xiv. STable 9.

STable 9. List of primers and annealing temperatures used for gene amplification.

Gene	Primer Name	Primer Sequence (5'→3')	Annealing Temperatures	Source
CYTB	Cytb1	ATT TGT CGA GAT GTR AAY TAY GG	50˚C	Hunter *et al*., 2008
	Cytb2	AAA TAT CAT TCN GGY TGR ATR TG		
COI	LCO1490	GGT CAA CAA ATC ATA AAG ATA TTG G	46˚C	Folmer et al., 1994
	HCO2198	TAA ACT TCA GGG TGA CCA AAA AAT C[illegible]		
	COFA79	TCT CCA CCA TAA AGA TAT TGG	49.5˚C	This study
	COR962	GCC GTG AAG TGT TCC TAA TC		
	COF575	TAT TAC TCC TTT CCC TCC CAG		
	COR1278	ACC GTT GAG ACC TAA GAA ATG C		
16S	16SarL	CGCCTGTTTATCAAAAACAT	47˚C	Palumbi, 1991
	16SbrH	CCGGTCTGAACTCAGATCACGT		
H3	H3aF	ATGGCTCGTACCAAGCAGACVGC	50-53˚C	Colgan et al., 1998
	H3aR	ATATCCTTRGGCATRATRGTGAC		
18S	18S1F	TAC CTG GTT GAT CCT GCC AGT AG	49˚C	Giribet et al. 1996
	18S5R	CTT GGC AAA TGC TTT CGC		
	18S3F	GTT CGA TTC CGG AGA GGG A	52˚C	
	18Sbi	GAG TCT CGT TCG TTA TCG GA		
	18Sa2.0	ATG GTT GCA AAG CTG AAA C	49˚C	
	18S9R	GAT CCT TCC GCA GGT TCA CCT AC		
28S	28SC1'	ACC CGC TGA ATT TAA GCA T	52˚C	Le et al. 1993<br>Vonnemann et al. 2005
	28SD3	GAC GAT CGA TTT GCA CGT CA		

xv. **STable 10.**

STable 10. Model selection per gene based on BIC within IQ-TREE ModelFinder.

Analysis	Partitioning Scheme	Substitution Model (IQ-TREE / BAYES)
Single Gene - H3	[H3 (codon1) + H3 (codon2)] + H3 (codon3)	(JC/JC) + (K2P/K2P)
Single Gene - CYTB	[CYTB (codon1) + CYTB (codon2)] + CYTB (codon3)	(HKY+F+I/HKY+F+I) + (TN+F+I/HKY+F+G4)
Single Gene - COI	COI (codon1) + COI (codon2) + COI (codon3)	(Tne+I+G4/K2P+I) + (F81+F+I/F81+F+I) + (Tn+F+G4/GTR+F+G4)
Single Gene - 16S	NA	(TIM3+F+G4/HKY+F+G4)
Single Gene - 18S	NA	(K2P+I/K2P+I)
Single Gene - 28S	NA	(TIM3+F+I/GTR+F+I)
Multi Gene	[COI (codon1)+CYTB (codon1)], + [COI (codon2)+CYTB (codon2)], + [COI (codon3)+CYTB (codon3)], + [16S+H3 (codon3)], + [18S+H3 (codon1)], + [28S+H3 (codon2)]	(Tim2e+1/K2P+I), + (HKY+F+I/HKY+F+I) + (TN+F+G4/GTR+G4) + (TIM3+F+G4/GTR+F+G4) + (K3P/K2P) + (TIM3+F+I/GTR+F+I)

xvi. STable 11.

STable 11. GenBank accession numbers for anchialine atyid taxa evaluated for divergence dating and stochastic mapping of salinity preferences. LS/S, Low Salinity/Stenohaline (<4.9 psu); HS/E, High Salinity/Euryhaline (5–37 psu).

Taxon		CO1	CYTB	16S	H3	18S	28S	Salinity	Reference
Halocaridina rubra	*Hawaii*	DQ917432, NC008413 KF437502-KF437509			HE800964	HE801015	FN995585	HS/E	Bailey-Brock and Brock, 1993
Halocaridinides fowleri	*Zanzibar*	KX844723	KX844723	KX844723	--	--	--	HS/E	Gurney, 1984
Halocaridinides trigonophthalma	*Japan*	--	--	--	FN995501	EF173849	FN995586_	HS/E	Fugino and Shokita, 1975
Stygiocaris lancifera	*W. Australia*	NC035404	NC035404	NC035404	HE800968	HE801019	FN995620	LS/S	Holthuis, 1960
Stygiocaris stylifera	*W. Australia*	KX844722	KX844722	KX844722	HE800969	HE801020	FN995622	LS/S	Holthuis, 1960
Typhlatya arfeae	*France*	KX844721	KX844721	KX844721	HE800975	HE801025	HE801045	HS/E	Jaume and Bréhier, 2005
Typhlatya consobrina	*Cuba*	KX844717	KX844717	KX844717	HE800979	HE801028	HE801048	LS/S	Sanz and Platvoet, 1996
Typhlatya dzilamensis	*Mexico*	KX844719	KX844719	KX844719	--	--	--	HS/E	Alvarez et al., 2005; This study
Typhlatya dzilamensis (TI013A)	*Mexico*	--	--	--	OM455426	OM458838	OM458884		Alvarez et al., 2005; This study
Typhlatya galapagensis (Isabella)	*Galapagos Islands*	KX844711	KX844711	KX844711	HE800966	HE801018	HE801038	HS/E	Monad and Cals, 1970
Typhlatya galapagensis (Santa Cruz)	*Galapagos Islands*	KX844718	KX844718	KX844718	HE800967	HE801017	HE801037	HS/E	Monad and Cals, 1970
Typhlatya garciai (Hunter)	*Caicos Island*	DQ863170	DQ900833	DQ863136	--	--	--	HS/E	Chace, 1942; Buden & Felder, 1975
Typhlatya garciai (Botello)	*Caicos Island*	HE800909	HE800955	HE801003	--	--	--	HS/E	Chace, 1942; Buden & Felder, 1975
Typhlatya garciai (Jurado)	*Cuba*	KX844720	KX844720	KX844720	--	--	--	LS/S	Sanz and Platvoet, 1995
Typhlatya iliffei	*Burmuda Island*	KX844710	KX844710	KX844710	HE800973	HE801023	HE801043	HS/E	Hart and Manning, 1981
Typhlatya miravetensis	*Spain*	LT608343	LT608343	LT608343	HE800974	HE801024	HE801044	LS/S	Sanz and Platvoet, 1995
Typhlatya mitchelli (TI008D)	*Yucatan Peninsula*	OM456519	OM455438	OM458914	OM455425	OM458837	OM458883	LS/S	Hobbs et al., 1997, This study
Typhlatya monae	*Dominican Republic*	KX844715	KX844715	KX844715	HE800976	HE801026		HS/E	Sanz and Platvoet, 1996
Typhlatya pearsei	*Yucatan Peninsula*	KX844709	KX844709	KX844709	--	--	--	LS/S	Hobbs et al., 1997, This study
Typhlatya pearsei (TP12)	*Yucatan Peninsula*	--	--	--	XXXXXX	XXXXXX	OM458890		Hobbs et al., 1997, This study
Typhlatya rogersi	*Ascension Island*	HE800908	--	HE801002	HE800977	HE801027	FN995628	HS/E	Chace and Manning, 1972
Typhlatya sp. A (TI157B)	*Mexico*	OM456521	XXXXXX	OM458916	OM455427	OM458839	OM458885	LS/S	This study
Typhlatya sp. B (mitchelli)	*Mexico*	KX844712	KX844712	KX844712	--	--	--	LS/S	This study
Typhlatya sp.	*Zanzibar*	KX844713	KX844713	KX844713	--	--	--	HS/E	Jurado et al., 2017, K. Melend, pers. comm.
Typhlatya taina	*Cuba*	KX844708	KX844708	KX844708	HE800980	HE801029	HE801049	LS/S	Estrada and Gómez, 1987
Typhlopatsa pauliani	*Madagascar*	KX844716	KX844716	KX844716	--	--	--	HS/E	Holthius, 1965
